# Transcriptome Remodeling in *Trypanosoma cruzi* and Human Cells during Intracellular Infection

**DOI:** 10.1371/journal.ppat.1005511

**Published:** 2016-04-05

**Authors:** Yuan Li, Sheena Shah-Simpson, Kwame Okrah, A. Trey Belew, Jungmin Choi, Kacey L. Caradonna, Prasad Padmanabhan, David M. Ndegwa, M. Ramzi Temanni, Héctor Corrada Bravo, Najib M. El-Sayed, Barbara A. Burleigh

**Affiliations:** 1 Department of Cell Biology and Molecular Genetics, University of Maryland, College Park, Maryland, United States of America; 2 Department of Immunology and Infectious Diseases, Harvard School of Public Health, Boston, Massachusetts, United States of America; 3 Center for Bioinformatics and Computational Biology, University of Maryland, College Park, Maryland, United States of America; Zentrum für Molekulare Biologie der Universität Heidelberg (ZMBH), GERMANY

## Abstract

Intracellular colonization and persistent infection by the kinetoplastid protozoan parasite, *Trypanosoma cruzi*, underlie the pathogenesis of human Chagas disease. To obtain global insights into the *T*. *cruzi* infective process, transcriptome dynamics were simultaneously captured in the parasite and host cells in an infection time course of human fibroblasts. Extensive remodeling of the *T*. *cruzi* transcriptome was observed during the early establishment of intracellular infection, coincident with a major developmental transition in the parasite. Contrasting this early response, few additional changes in steady state mRNA levels were detected once mature *T*. *cruzi* amastigotes were formed. Our findings suggest that transcriptome remodeling is required to establish a modified template to guide developmental transitions in the parasite, whereas homeostatic functions are regulated independently of transcriptomic changes, similar to that reported in related trypanosomatids. Despite complex mechanisms for regulation of phenotypic expression in *T*. *cruzi*, transcriptomic signatures derived from distinct developmental stages mirror known or projected characteristics of *T*. *cruzi* biology. Focusing on energy metabolism, we were able to validate predictions forecast in the mRNA expression profiles. We demonstrate measurable differences in the bioenergetic properties of the different mammalian-infective stages of *T*. *cruzi* and present additional findings that underscore the importance of mitochondrial electron transport in *T*. *cruzi* amastigote growth and survival. Consequences of *T*. *cruzi* colonization for the host include dynamic expression of immune response genes and cell cycle regulators with upregulation of host cholesterol and lipid synthesis pathways, which may serve to fuel intracellular *T*. *cruzi* growth. Thus, in addition to the biological inferences gained from gene ontology and functional enrichment analysis of differentially expressed genes in parasite and host, our comprehensive, high resolution transcriptomic dataset provides a substantially more detailed interpretation of *T*. *cruzi* infection biology and offers a basis for future drug and vaccine discovery efforts.

## Author Summary

In-depth knowledge of the functional processes governing host colonization and transmission of pathogenic microorganisms is essential for the advancement of effective intervention strategies. This study focuses on *Trypanosoma cruzi*, the vector-borne protozoan parasite responsible for human Chagas disease and the leading cause of infectious myocarditis worldwide. To gain global insights into the biology of this parasite and its interaction with mammalian host cells, we have exploited a deep-sequencing approach to generate comprehensive, high-resolution transcriptomic maps for mammalian-infective stages of *T*. *cruzi* with the simultaneous interrogation of the human host cell transcriptome across an infection time course. We demonstrate that the establishment of intracellular *T*. *cruzi* infection in mammalian host cells is accompanied by extensive remodeling of the parasite and host cell transcriptomes. Despite the lack of transcriptional control mechanisms in trypanosomatids, our analyses identified functionally-enriched processes within sets of developmentally-regulated transcripts in *T*. *cruzi* that align with known or predicted biological features of the parasite. The novel insights into the biology of intracellular *T*. *cruzi* infection and the regulation of amastigote development gained in this study establish a unique foundation for functional network analyses that will be instrumental in providing functional links between parasite dependencies and host functional pathways that have the potential to be exploited for intervention.

## Introduction

**Fig 1:**
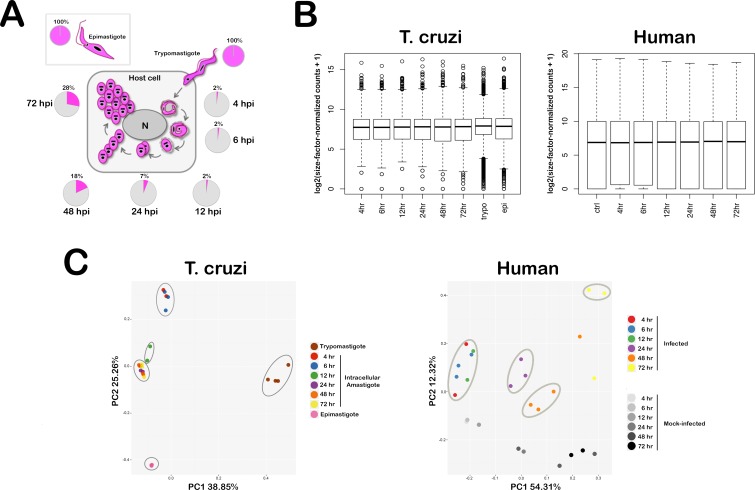
Simultaneous interrogation of parasite and host transcriptomes. **(A) Intracellular *T*. *cruzi* life cycle and sample collection scheme.** Extracellular *T*. *cruzi* trypomastigotes actively penetrate mammalian cells where they receive cues to differentiate into amastigote forms that replicate in the host cell cytoplasm for 3–5 days, beginning at ~22 hpi with a doubling time of ~12 hr. Amastigote division ceases on day 4 or 5 post-infection and parasites differentiate back into trypomastigotes that rupture the host cell to initiate a new cellular infection cycle. For RNA-Seq analysis, total RNA was isolated from axenic *T*. *cruzi* epimastigotes (insect vector stage), extracellular trypomastigotes and from amastigote-containing human fibroblast monolayers at 6 time points spanning 4–72 hpi. Pie charts indicate the proportion of mapped sequence reads assigned to the parasite (pink) or human (grey). (**B) Distribution of global gene expression levels in a representative subset of *T*. *cruzi* and human samples.** Box plots showing comparisons of the distribution of per-gene counts (log2 counts per million with an offset of 1) normalized for sequencing library size. The ends of the whiskers represent the lowest datum still within 1.5 interquartile range (IQR) of the lower quartile, and the highest datum still within 1.5 IQR of the upper quartile. Genes with extremely high or low expression levels are shown as open circles above and below the whiskers, respectively. **(C) Principal component analysis plots of global transcriptome profiles.** Principal component analysis (PCA) plot for RNA-Seq data with *T*. *cruzi* and human samples plotted separately. The two first principal components (PC1 and PC2) are plotted with the proportion of variance explained by each component next to the axes labels. Each sample is represented by a dot and the color label corresponding to the sample group, such as the number of hours (hr) post-infection.

The kinetoplastid protozoan parasite *Trypanosoma cruzi* is the etiologic agent of human Chagas disease. This parasite has a complex life cycle that involves hematophagous triatomine insects as vectors for transmission and a broad range of mammalian hosts including extensive domestic animal and sylvatic reservoirs [1]. Epimastigote forms of the parasite proliferate in the midgut of the insect vector and give rise to non-dividing, mammalian-infective metacyclic trypomastigotes that are shed in the feces of blood-feeding triatomine bugs and initiate infection in the vertebrate host. *T*. *cruzi* trypomastigotes actively penetrate a wide range of nucleated cell types, become enveloped in an acidified lysosome-like compartment [2] where they receive signals to differentiate into amastigotes [3]. Differentiating parasites gradually escape the lysosomal vacuole [4] and proliferate as amastigotes in the host cell cytosol for 3–5 days (Fig 1A) before differentiating back into trypomastigotes (referred to as tissue or tissue culture trypomastigotes to distinguish these from metacyclic trypomastigotes), which are released into the extracellular space/medium upon host cell lysis. Motile trypomastigotes disseminate infection via the lymphatics and bloodstream to distal sites where they undergo further cycles of intracellular multiplication, egress and reinvasion. Thus, at several key points in its life cycle, *T*. *cruzi* undergoes developmental reprogramming to adapt to different hosts and variable niches within hosts, however the mechanisms governing these adaptive processes are not well defined.

Cellular differentiation is controlled at multiple levels including, for most eukaryotic cells, initiation of gene transcription (eg. [5, 6]). In trypanosomatids discriminatory mechanisms for the initiation of transcription at individual loci is largely absent. Most protein-coding genes lack promoters and are transcribed as long polycistronic units that are processed into individual mRNAs [7–10]. Consequently, trypanosomes rely on post-transcriptional processes such as mRNA stability, translational efficiency and post-translational modification to coordinate developmental transitions and other adaptive responses encountered throughout their complex life cycles [11–15]. Despite the recent emphasis on mRNA translation efficiency as a primary regulator of protein abundance in trypanosomatids [13, 16–18] and across eukaryotes more generally [19], there is strong evidence for the existence of post-transcriptionally generated mRNA regulons in *Trypanosoma brucei* and *Leishmania* that coordinate major developmental shifts in these organisms [20–23].

As with other eukaryotes, mRNA stability and translational efficiency are influenced by *trans*-acting factors (RNA-binding proteins: RBPs) that interact with *cis*-acting regulatory elements in the untranslated regions of trypanosomatid mRNAs (recently reviewed in [15, 24]). Because *trans*-acting factors regulate multiple mRNAs in a combinatorial fashion [25, 26], it has been challenging to identify *cis*-acting and *trans*-acting elements that are associated with the expression of functionally-regulated trypanosomatid genes [27]. However, a growing number of examples link candidate RBP expression levels with the modulation of mRNA subsets (eg. [15]). Indeed, an entire cellular differentiation program was shown to be triggered by the over expression of a single RBP in African trypanosomes [28]. Functional *cis*-acting elements have been identified in a number of *T*. *cruzi* transcripts and associated with the regulation of expression in this organism [29, 30] including sets of developmentally-regulated [30–33] and functionally-related [34] genes. Although suggestive of the existence of mRNA regulons in *T*. *cruzi*, high-resolution transcriptomic data are needed to relate dynamic changes in parasite gene expression to functional adaptation on a global scale. Here, we exploit deep sequencing and informatics approaches to construct high-resolution transcriptome maps for three main *T*. *cruzi* lifecycle stages and include the simultaneous capture of parasite and host transcriptional responses during an intracellular infection of human fibroblasts by *T*. *cruzi*. With this approach, we gain deeper insights into the biology of *T*. *cruzi* with an emphasis on intracellular infection and conclude that transcriptome remodeling is required to alter the ‘blueprint’ upon which major developmental transitions are based.

## Results/Discussion

### Simultaneous capture of *T*. *cruzi* and human host cell transcriptomes by RNA-seq

To capture the global transcriptomic response associated with the establishment and maintenance of intracellular *T*. *cruzi* infection, RNA was isolated from low passage primary human foreskin fibroblasts (HFF) infected with tissue culture-derived *T*. *cruzi* Y strain trypomastigotes, and from mock-infected cells, at 4, 6, 12, 24, 48 and 72 hours post-infection (hpi) (Fig 1A). RNA was also generated from extracellular trypomastigotes and from axenically cultured log-phase *T*. *cruzi* epimastigotes for comparative purposes. Two to four independent biological replicates were sequenced for each condition generating 2.7 billion high quality reads from 35 samples (S1 Table) that were subsequently processed through our RNA-Seq and data analysis pipeline (S1 Fig). Sequence reads generated from *T*. *cruzi*-infected cell samples were resolved by mapping pre-processed reads against *T*. *cruzi* [35] and human hg19 reference genomes using the Tophat aligner program [36] (S2 and S3 Tables). The well-documented differences in transcriptional regulation between trypanosomes and humans [7, 8, 10] were reflected in the distributions of the log2-transformed and size-factor-normalized gene counts for both species (Fig 1B and S2 Fig). As expected, the fraction of total reads mapping to the *T*. *cruzi* genome from the mixed host-parasite read pool increased over time as intracellular amastigote replication ensued (Fig 1A). It is worth noting that due to the stringency imposed during mapping (≤ 2 mismatches allowed/read) and the necessity to map *T*. *cruzi* Y strain sequences against a heterologous (CL Brener Esmeraldo) genome [35], the depth of coverage of the *T*. *cruzi* transcriptome at each stage (S1 Table) is most certainly underestimated. Despite this limitation, the demonstrated ability to resolve parasite and human sequences from a mixed read pool, and to obtain a high level of coverage of the *T*. *cruzi* transcriptome, bodes well for future transcriptomic analyses of the *T*. *cruzi* infection process *in vitro* and *in vivo*, particularly as whole genome sequence information for additional *T*. *cruzi* strains become available (eg. [37, 38]).

The overall reproducibility and experimental variation between similarly generated independent samples was evaluated with Pearson correlation (S3 Fig) and median pairwise correlation analyses for *T*. *cruzi* (S4 Fig) and human (S5 Fig) samples. For *T*. *cruzi*, biological replicates corresponding to each of the parasite developmental stages were highly correlated (S3A and S4 Fig), with the intracellular stages (from 4–72 hpi) exhibiting greater similarity to each other than to either of the extracellular stages (trypomastigotes and epimastigotes) (S3A Fig). The human transcriptome samples also displayed a high level of correlation between biological replicates (S3B and S5 Fig). One exception (“4hr2”, HPGL0111), identified as an outlier in a more systematic median pairwise correlation analysis (S6 Fig), was removed from downstream analysis (S3B and S5 Figs).

To investigate general trends in the data while identifying and quantifying batch effects, principal component analysis (PCA) was carried out (Fig 1C) as well as hierarchical clustering of all parasite (S7A Fig) and human (S7B Fig) samples. PCA plots reveal a high degree of similarity between biological replicates for both the *T*. *cruzi* and human samples (Fig 1C). For *T*. *cruzi*, the extracellular parasite stages (trypomastigotes and epimastigotes) were well separated from each other and displayed very tight clustering within each group (Fig 1C; *T*. *cruzi*). The intracellular stages grouped according to their maturation status, with nascent amastigotes (4 and 6 hr) clustering together and well separated from the mature replicative amastigote stages (24, 48, 72 hr) with the 12 hr amastigotes in between (Fig 1C; *T*. *cruzi*). A similar trend is observed for the human data (Fig 1C; Human). Parasite-infected HFF samples are well separated from uninfected samples (PC2) and the early infection time points (4–12 hpi) clustered together (Fig 1C; Human). The later infection time points were more loosely clustered with outliers observed at 48 and 72 hpi (Fig 1C; Human). Notably, the transcriptome of uninfected fibroblasts changed considerably with time in culture (Fig 1C; Human PC1) underscoring the necessity to include mock-infected controls for each infection time course for direct comparison, as we have done here. Consistent with the PCA results, the unsupervised hierarchical clustering of *T*. *cruzi* samples (S7 Fig) labeled both by biological group and experimental batch date segregated trypomastigote, epimastigote and intracellular amastigote samples into distinct clusters. The partitioning of immature (4, 6, 12 hpi) and mature (24, 48 and 72 hpi) intracellular developmental stages of *T*. *cruzi* is also evident (S7A Fig). A similar partitioning of infected human cell samples into early (4, 6 and 12 hpi), mid (24 and 48 hpi) and late (72 hpi) time points suggest distinct phases of the host cell response to parasite infection (S7B Fig).

To extract biologically meaningful inferences from our expression data, lists of differentially expressed genes (DEGs) were constructed from pairwise comparisons of parasite or human expression data (S4 Table). As a large number of genes survived this initial cut-off (q-value < 0.05) a second filter (log_2_fold-change ≥1.0) was imposed to identify gene expression changes with the highest potential impact (S4 Table) for *T*. *cruzi* developmental stages (S5 Table) and human fibroblasts at different stages of *T*. *cruzi* infection with matched controls (S7 Table). Because of frequent gene duplication and the presence of several expanded gene families in the *T*. *cruzi* genome, the parasite DEG data (S5 Table) was filtered to remove all but a single representative of each paraloguous group (S6 Table). DEG information for *T*. *cruzi* (S6 Table) and human fibroblasts (S7 Table) were used for downstream Gene Ontology (GO) enrichment analysis [39, 40] and K-means clustering [41] (S1 Fig). Highlights from this collective analysis are presented and discussed in the context of the relevant biology of *T*. *cruzi*-host cell interactions in the following sections.

### Transcriptomic signatures mirror biological features of *T*. *cruzi* developmental stages

Steady-state transcriptome data was generated for three distinct *T*. *cruzi* life stages with a more comprehensive analysis of intracellular amastigote development in human fibroblasts (Fig 1A). Focusing on the most disparate samples first, i.e. those derived from distinct developmental stages of *T*. *cruzi*: epimastigotes, trypomastigotes and intracellular amastigotes (24 hpi), we observed ≥2 fold differences in steady state transcript abundance for ~2000 to ~3500 parasite genes (S4 and S5 Tables) or between ~1500–2600 after correcting for paralogues (S4 Table). Thus, as a conservative estimate, we find stage-regulated changes in transcript abundance to occur for ~15–30% of the predicted protein-coding genes in the *T*. *cruzi* genome contrasting with a previous estimate of >50% derived from comparative microarray hybridization analysis of *T*. *cruzi* life stages [42].

Due to the nature of polycistronic transcription in *T*. *cruzi* [7–9] few parasite transcripts are expected to exhibit strict stage-specificity ie. detectable in one life stage and undetectable in others (Fig 1B). Despite this, we find a subset of *T*. *cruzi* genes (between ~135 and 350) to be over-represented at the transcript level in a single life cycle stage as compared to the other two developmental stages compared in this study (S8 Table). Adding weight to this approach, we observed the expected stage-selective expression of δ- and β-amastins in amastigotes and epimastigotes respectively (S8 Table). With the high proportion of hypothetical genes (>50% in each gene list; S8 Table) we were not able to identify significantly enriched GO terms associated with parasite stage-enriched genes. Nonetheless, some general observations offer insight into lifestyle differences among these different parasite stages. For example, trypomastigotes uniquely express a number of protein kinases and an intermediate filament-binding trichohyalin-like protein [43], which may reflect specialized capacities linked to host cell recognition, signaling and invasion by this parasite life stage [44–46]. Epimastigotes express twice as many genes in a stage-selective manner as the other parasite life stages (S8 Table), many of which encode metabolic enzymes (eg. pentose phosphate pathway, amino acid metabolism) or proteins involved in nutrient acquisition (eg. transporters of hexose sugars, nucleosides, folate/pteridine and amino acids). In contrast, amastigotes display elevated expression of cation transporters including (TcCLB.509197.39) which is upregulated early in amastigote development (by 6 hpi) with mRNA abundance reaching very high levels by 12 hpi (log_2_fold-change 4.2; ~20-fold increase) to become the most highly expressed amastigote gene at 72 hpi (~35-fold increase) (S5 Table) as confirmed by qRT-PCR (S9 Fig). Another cation transporter gene (TcCLB.507527.50) that displays preferential expression in amastigotes at 24 hpi (S5 and S8 Tables) bears homology to the ferrous iron transporter characterized in *Leishmania amazonensis* [47], the expression of which is intimately coupled to amastigote development in this parasite. Thus, selective cation transporter expression, and potentially the need for iron uptake, may be a common feature of amastigote development and intracellular maintenance in both *Leishmania* and *T*. *cruzi*.

By virtue of their critical role in regulating gene expression in trypanosomatids [15], RNA-binding proteins (RBPs) represent another class of genes for which parasite stage-selective expression information is of interest. Only the RBPs that exhibit differential expression during the intracellular infection process are highlighted (S10A Fig). *T*. *cruzi* trypomastigotes selectively express two RBPs, RBP5 (TcCLB.511127.10) and RBP6 (TcCLB.506693.30) (S8 Table). While RBP5 has yet to be characterized, RBP6 has emerged as a critical regulator of metacyclogenesis in *T*. *brucei* [28]. Therefore, stage-specific expression of the orthologuous gene in *T*. *cruzi* trypomastigotes suggests that TcRBP6 may exert important stage-specific functions in the non-dividing, cell-invasive forms of this parasite as well. While several RBPs exhibit transient and relatively low level increases in expression in developing amastigotes, a notable exception to this trend is TcCLB.504005.6 (S5 Table), for which mRNA abundance tracks with increasing intracellular amastigote numbers (S10 Fig). It is tempting to speculate that this particular RBP may participate in regulating processes critical to late stage amastigote growth or the next phase of the infection cycle that involves amastigote to trypomastigote conversion starting at ~96–120 hpi. Genome-scale dynamic mRNA expression data for RNA-binding proteins provides new and valuable information regarding the life cycle stage at which these important *trans*-acting mRNA regulatory factors are likely to act.

### Extensive transcriptome remodeling in *T*. *cruzi* accompanies adaptation to life inside the mammalian host cell

To gain global insights into the intracellular *T*. *cruzi* infection process, a focused transcriptomic analysis was performed to capture dynamic changes in *T*. *cruzi* mRNA abundance as the parasite established intracellular residence in mammalian host cells (Fig 1A). Extensive remodeling of the *T*. *cruzi* transcriptome was observed within the first 4 hours of trypomastigote invasion of human fibroblasts (2790 DEGs, S4 and S5 Tables), corresponding to the dramatic shift in environment and initiation of the amastigote differentiation program. More modest changes in *T*. *cruzi* transcript abundance occurred as amastigote development and maturation progressed over the next 20 hours of the infection cycle (644 DEGs in the 4–24 hpi interval) (S4 and S5 Tables). Then, once the amastigotes entered into replicative phase of the intracellular infection cycle (~22 hpi), few additional changes in the steady state transcriptome were detected (43 DEGs emerge in the 24–72 hpi interval; S4 and S5 Tables). While it is conceivable that a failure to detect additional DEGs at this stage is due to the masking of subtle transcriptome dynamics in asynchronously replicating amastigote populations, an alternative interpretation of this observation is that widespread transcriptome remodeling is only required to launch the *T*. *cruzi* amastigote differentiation program. Upon completion of the developmental switch, other important aspects of amastigote biology, such as nutrient acquisition and cell cycle regulation, are likely controlled by mechanisms other than mRNA stability. While we lack definitive data for this prediction, our observations align well with documented global gene expression patterns in related trypanosomatids, *T*. *brucei* [12, 48] and *Leishmania donovani* [11, 13, 23], where mRNA stability is cited as playing a more prominent role in early parasite development and both translation efficiency and post-translational modification acting as the main regulatory processes that control homeostatic functions [11, 13, 16–18, 23, 48].

### Signatures of early *T*. *cruzi* amastigote development in mammalian cells

If transcriptome remodeling is required to generate a new blueprint to guide developmental transitions in *T*. *cruzi*, it follows that associated changes in morphology and functionality should be reflected in corresponding transcriptomic signatures. The capture of dynamic changes in parasite mRNA abundance over a time course of infection in human fibroblasts provides an opportunity to derive biological inferences based on differential expression patterns and to compare these with known aspects of *T*. *cruzi* amastigote biology. Focusing first on the early phase of intracellular infection by *T*. *cruzi* where the greatest number of DEGs was detected, we observe several expected features of the trypomastigote to amastigote transition in the transcriptome changes. These include: (1) rapid downregulation of transcripts encoding major polymorphic surface protein classes (trans-sialidases, mucins, MASPs and gp63) (S4 and S5 Tables Trypo vs Ama4), some of which have been implicated in host recognition, signaling and immune evasion [45, 49–51]; (2) reduced expression of genes involved in flagellar assembly and motility (within 4 hpi) (S5 and S9 Tables) coincident with dramatic shortening of the single *T*. *cruzi* flagellum; and (3) increased abundance of transcripts encoding the amastigote-specific surface protein, δ-amastin (S5 Table) [31, 52]. Consistent with the plasma membrane remodeling that is expected during trypomastigote to amastigote differentiation, we also observe significantly increased transcript levels for GPI-inositol deacylase (TcCLB.510289.40) [53], membrane-bound/secreted phospholipase A1 [54] (TcCLB.509011.90; S5 Table) and a surface-localized phosphatidylinositoI-phospholipase C (PI-PLC) (TcCLB.504149.160) [55] (S5 Table). Although central to parasite plasma membrane remodeling during differentiation [56], amastigote surface lipases are also well positioned to facilitate breakdown of the parasitophorous vacuole alongside the activity of a secreted hemolysin [57], analogous to the mechanism of vacuole egress by the intracellular bacterial pathogen, *Listeria monocytogenes* [58]. Moreover, once *T*. *cruzi* amastigotes become cytosolically-localized in mammalian cells there are ample opportunities for parasite surface and secreted/released products to interact with host molecules and to modulate host functions during the course of infection. In this regard, *T*. *cruzi* amastigote phospholipase A1 can be considered a parasite-derived effector protein given that its expression is associated with perturbations in host cell phospholipid metabolism [59] and the activation of host protein kinase C [60].

In addition to remodeling at the plasma membrane during the early trypomastigote to amastigote transition, indicators of signaling pathway retooling also emerge in the transcriptome data (S5 Table; Trypo vs Ama4). For example, a number of predicted protein kinases and phosphatases are differentially expressed in the parasite shortly after trypomastigote entry into mammalian host cells (S5 Table; Trypo vs Ama4) including the previously characterized farnesylated protein tyrosine phosphatases (TcCLB.506743.130; TcCLB.506743.110) [61]. Consistent with the recognized role of cyclic AMP in *T*. *cruzi* differentiation processes [62], we also observe developmental regulation of central components of the cAMP-dependent signaling pathway, such as receptor-type adenylate cyclases (eg. TcCLB.511043.60; TcCLB.428999.20; TcCLB.507467.10), cAMP-dependent protein kinase A (TcCLB.509805.10; TcCLB.506227.150) and cAMP-dependent phosphodiesterases (eg. TcCLB.508277.100; TcCLB.506625.80) (S5 Table; Trypo vs Ama4). In the broader context of sensory detection and signal transduction, it is worth noting that ‘ciliary and flagellar motility’ emerges as an enriched GO term associated with mature *T*. *cruzi* amastigotes (≥48 hpi) when compared to immature amastigote stages (S9 Table; eg. Ama4 vs Ama48). This signature is driven by the increase in mRNA abundance for several flagellum-associated protein coding genes in mature amastigotes after their initial decline during the initial stages of amastigote development (S10B Fig). The expression of the calcium-sensing FCaBP family members [63] in intracellular *T*. *cruzi* amastigotes suggests that the minimal amastigote flagellum may engage in sensory functions in the intracellular life stages, as suggested for *Leishmania* amastigotes [64]. As little is known regarding the mechanism(s) by which *T*. *cruzi* parasites detect and integrate sensory information, particularly for the intracellular mammalian-infective stages, the availability of high-resolution mRNA expression data for *T*. *cruzi* intracellular stages (S5 and S8 Tables) opens the door to discovery of additional parasite molecules that function in a sensory or signaling capacity including the many predicted protein kinases and phosphatases encoded in the *T*. *cruzi* genome [65] that have yet to be characterized.

Consistent with a period of rapid remodeling during early amastigote development in which proteins and membranes are expected to undergo extensive turnover, Gene Ontology enrichment analysis identified ribosomal RNA processing (GO:0006364), protein translation (GO:0006412) and protein folding (GO:0006457) as significantly enriched GO terms associated with nascent and developing amastigotes (S9 Table; Trypo vs Ama4-Ama12). Despite the fact that nascent intracellular amastigotes will not undergo a first round of replication for another ~20 hours, preparation for the eventuality of cell doubling is already evident in the transcriptome of early amastigote stages. Consistent with the projected increase in nucleic acid synthesis, nucleoside transporters (TcCLB.506203.10; TcCLB.506773.50) and enzymes involved in pyrimidine synthesis (eg. orotidine-5-phosphate decarboxylase; TcCLB.508373.29) and purine salvage are upregulated in immature amastigotes (S5 Table). Genes encoding enzymes in the guanine branch of the purine salvage pathway [66] are selectively upregulated in intracellular amastigotes as compared to trypomastigotes (eg. guanine deaminase: TcCLB.504431.100; inosine 5' monophosphate dehydrogenase (IMPDH) TcCLB.507211.40; TcCLB.511351.9; XPRT; GMP synthase: TcCLB.508085.10; GMP reductase; TcCLB.506519.130). In contrast, enzymes associated with the adenine branch are preferentially expressed in trypomastigotes over amastigotes: eg hypoxanthine-guanine phosphoribosyltransferase: TcCLB.506457.30; adenine phosphoribosyltransferase: TcCLB.508207.74; adenylsuccinate synthetase: TcCLB.508731.60). This observation raises the possibility that flux through the purine salvage pathway is tuned to the different environments encountered by *T*. *cruzi* life stages.

**Fig 2:**
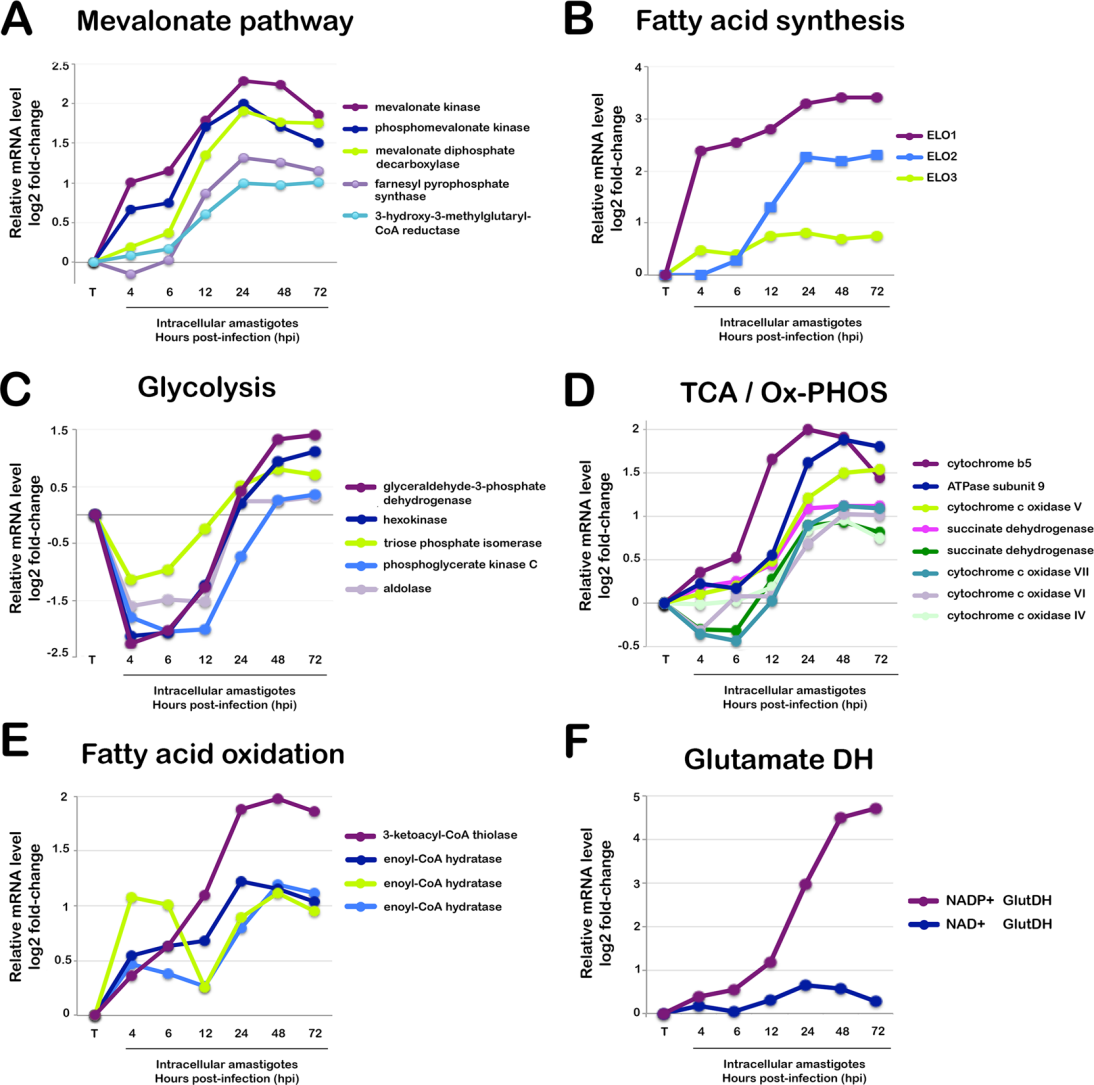
Temporal expression of metabolic pathway genes in mammalian-infective stages of *T*. *cruzi*. Relative mRNA expression of selected genes in intracellular *T*. *cruzi* amastigote stages (4–72 hpi) compared to extracellular trypomastigotes (**T**). Genes in the following metabolic pathways are highlighted: (**A) Mevalonate pathway**: mevalonate kinase (TcCLB.436521.9), mevalonate diphosphate decarboxylase (TcCLB.507993.330), squalene monooxygenase (TcCLB.509589.20), farnesyl pyrophosphate synthase (TcCLB.508323.9), 3-hydroxy-3-methylglutaryl-CoA reductase (TcCLB.509167.20). **(B) Fatty Acid Synthesis:** fatty acid elongase 1 (ELO1) (TcCLB.506661.30), fatty acid elongase 2 (ELO2) (TcCLB.506661.20), fatty acid elongase 3 (ELO3) (TcCLB.506661.10). **(C) Glycolysis:** glyceraldehyde-3-phosphate dehydrogenase (TcCLB.506943.60); hexokinase (TcCLB.508951.20), triosephosphate isomerase (TcCLB.508647.200), phosphoglycerate kinase (TcCLB.511419.40), aldolase (TcCLB.504163.40). (**D) Tricarboxylic Acid / Oxidative Phosphorylation (TCA/Ox-PHOS):** cytochrome b5 (TcCLB.506773.44), ATPase subunit 9 (TcCLB.503579.70), cytochrome c oxidase subunit V (TcCLB.510565.30), succinate dehydrogenase 11 (TcCLB.504035.84), succinate dehydrogenase 6 (TcCLB.507091.30), cytochrome c oxidase subunit VII (TcCLB.509233.150), cytochrome c oxidase subunit VI (TcCLB.511145.10), cytochrome c oxidase subunit IV (TcCLB.506529.360). (**E) Fatty Acid Oxidation:** 3-ketoacyl-CoA thiolase (TcCLB.510507.20), enoyl-CoA hydratase/isomerase (TcCLB.511529.170); enoyl-CoA hydratase, mitochondrial (TcCLB.508185.10) enoyl-CoA hydratase/isomerase (TcCLB.510997.40). **(F) Glutamate Dehydrogenases (DH):** NADP+-GlutDH (TcCLB.507875.20), NAD+ GlutDH (TcCLB.509445.39). All values are reported as log2 fold-change of difference between expression at in the trypomastigote stage and intracellular stages as reported in S5 Table.

The anticipated demand for lipid precursors to support membrane synthesis in *T*. *cruzi* amastigotes is mirrored by the enrichment of GO functions associated with isoprenoid (GO:0008299), sterol (GO:0006696) and fatty acid (GO:0006633) synthesis at early stages of amastigote development (S9 Table). Specifically, several enzymes in the mevalonate pathway are upregulated (Fig 2A) as are the first two enzymes in the fatty acid synthesis/elongation pathway [67] ELO1 (TcCLB.506661.30) and ELO2 (TcCLB.506661.20) (Fig 2B and S5 Table). Combined, these observations indicate that intracellular *T*. *cruzi* amastigotes generate sterols and fatty acids *de novo* to support replication and membrane homeostasis. Despite its biosynthetic capacity, *T*. *cruzi* may opt to scavenge some lipids or precursors from its host cell as seen with other parasites [68–70]. It is currently unclear if or how *T*. *cruzi* amastigotes balance *de novo* synthesis of macromolecular precursors with uptake from the host cell.

### Metabolic adaptation during intracellular *T*. *cruzi* development

**Fig 3:**
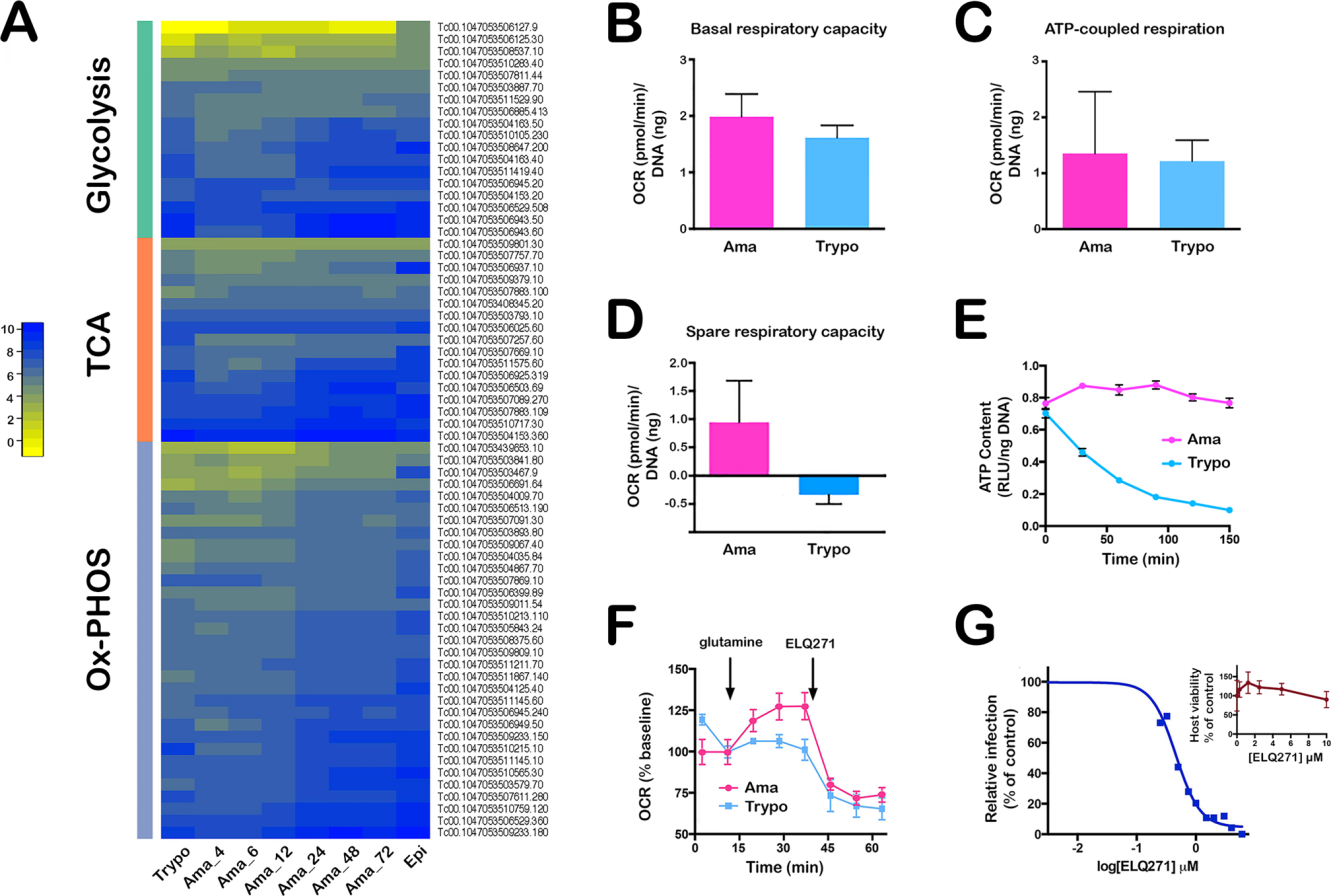
Validation of predicted metabolic features of *T*. *cruzi* developmental stages. **(A)** Heatmap of expression values of annotated *T*. *cruzi* genes predicted to function in intermediary metabolism with a focus on glycolysis, TCA cycle and Ox-PHOS. **(B)** Calculated basal respiratory capacity **(C)** ATP-linked respiration and **(D)** spare-respiratory capacity of extracellular trypomastigotes and isolated intracellular amastigotes (60 hpi) as pmol of oxygen consumed per min (oxygen consumption rate; OCR) normalized to *T*. *cruzi* DNA (ng) per well. **(E)** ATP content measured in isolated trypomastigotes and amastigotes in KHB buffer without a consumable carbon source at time points indicated. **(F)** OCR response to glutamine (10 mM) and ELQ271 (10 μM) in isolated *T*. *cruzi* trypomastigotes and amastigotes. **(G)** Dose-dependent inhibition of *T*. *cruzi* amastigote growth in HFF following addition of ELQ271 at 18 hpi and relative infection measured at 72 hpi. Host cell viability and growth (inset) is unaffected by the compound over the course of the assay. Graphs shown are representative of 3 independent experiments.

Intermediary metabolism has been of interest to biochemists in the *T*. *cruzi* field for more than fifty years (e.g. [71, 72]). *T*. *cruzi*, like its trypanosomatid relatives, has a partially compartmentalized glycolytic pathway [73] and a non-canonical TCA cycle is predicted [74]. All major *T*. *cruzi* life stages exhibit the capacity for oxidative phosphorylation [75, 76]. Although developmental differences in energy metabolism have been documented for *T*. *cruzi* (e.g. [77, 78]), the specific impact of mammalian host cell colonization on parasite and host bioenergetics remains a poorly understood aspect of the host-parasite relationship. Here, we report dynamic changes in the expression of *T*. *cruzi* genes involved in energy metabolism as trypomastigotes establish intracellular infection in mammalian cells (Fig 3A). Highlighted are glycolytic enzymes (Fig 2C) and components of the mitochondrial electron transport chain (Fig 2D) that exhibit biphasic responses during infection. *T*. *cruzi* transcripts encoding glycolytic enzymes were rapidly repressed in nascent amastigotes as compared to trypomastigotes (4–12 hpi) consistent with an earlier report that intracellular amastigotes do not take up hexose sugars [79]. However, transcript levels corresponding to a subset of glycolytic genes rebound in mature *T*. *cruzi* amastigotes (Fig 2C), with the emergence of ‘Glycolysis’ as an enriched GO term (GO:0006096) at this stage (>24 hpi) as compared to immature amastigotes (eg. Ama4) (S9 Table) suggesting that replicative amastigote stages likely retain some capacity for glycolysis inside the mammalian host cell.

Genes encoding enzymes involved in mitochondrial oxidative phosphorylation are also upregulated in the intracellular replicative stages of *T*. *cruzi* as compared to trypomastigotes (Fig 2D; Fig 3A) suggesting that the respiratory capacity differs for these two parasite life stages. A mitochondrial stress test was performed to test this prediction. In line with early comparative studies of mitochondrial respiratory capacity in *T*. *cruzi* life cycle stages [75, 80], basal respiration (Fig 3B) and ATP-coupled respiration (Fig 3C) were found to be similar for trypomastigotes and amastigotes. In contrast, the bioenergetic properties of these two parasite life stages diverged significantly at the level of mitochondrial spare respiratory capacity (SRC) (Fig 3D), an indicator of the potential of a cell to respond to sudden increases in energy demand [81]. In repeated measurements, isolated intracellular amastigotes displayed measurable mitochondrial reserve capacity whereas trypomastigotes had none (Fig 3D). Furthermore, trypomastigotes failed to maintain ATP levels in the absence of exogenous carbon, whereas homeostatic mechanisms to preserve cellular ATP levels were evident in amastigotes (Fig 3E). We hypothesize that mitochondrial reserve capacity may be important for *T*. *cruzi* amastigotes in the context of cell/tissue infection to provide a buffer against environmental stressors such as oxidative stress (e.g. [82]). A key short-term regulator of spare respiratory capacity in cells is cytochrome c oxidase (mitochondrial complex IV) [83]. Several cytochrome c oxidase subunits are more highly expressed at the transcript level in mature amastigotes as compared to trypomastigotes (Fig 2D and S5 Table). Differences in expression of this enzyme complex may contribute to observed homeostatic differences and mitochondrial SRC between these life stages. In the related organisms, *T*. *brucei* and *Leishmania*, cytochrome c oxidase subunit expression correlates with mitochondrial respiration rates, ATP production, parasite replication and virulence [84–86]. In this light, it would be interesting to probe the relationship between cytochrome c oxidase activity, mitochondrial spare respiratory capacity and *T*. *cruzi* strain-dependent differences in host infectivity and virulence.

The main carbon sources that fuel energy production in intracellular *T*. *cruzi* amastigotes are not definitively known. It is assumed that glucose is limiting in the host cell cytosol and that intracellular amastigotes rely on uptake of amino acids and fatty acids for energy [42, 87]. Our transcriptomic data generally support this projected trend with the anticipated increase in expression of fatty acid oxidation genes (Fig 2E) and of several amino acid permeases (S5 and S10 and S8 Fig cluster 5) during amastigote development in mammalian cells. In addition, glutamate dehydrogenase (GlutDH) was found to be highly expressed in replicative *T*. *cruzi* amastigote stages as compared to trypomastigotes (Fig 2F and S5 Table). GlutDH exerts an important anapleurotic function by converting glutamate to α-ketoglutarate that feeds into the TCA cycle to replenish intermediates diverted to biosynthetic functions. *T*. *cruzi* has two different glutamate dehydrogenase activities: one NAD+-linked [88] and the other NADP+-linked [89]. Both activities are expressed in epimastigotes [90]; also reflected in our mRNA expression analysis (S5 Table). However, only the NADP+-linked enzyme (TcCLB.507875.20) is upregulated in intracellular amastigotes (Fig 2F). The significance of this finding is unknown but suggests a degree of specialization for these enzymes in the two main replicative *T*. *cruzi* life stages. Consistent with higher GlutDH expression in *T*. *cruzi* amastigotes, we find that exogenous glutamine drove higher oxygen consumption rates (OCR) in isolated amastigotes as compared to trypomastigotes (Fig 3F). Oxidative phosphorylation is inhibited in both parasite life cycle stages by ELQ271 (Fig 3F), an endochin-like quinolone that selectively inhibits mitochondrial complex III activity of apicomplexan parasites over mammalian cells [91–93]. We further show that intracellular *T*. *cruzi* amastigote growth is inhibited by ELQ271 in a dose-dependent manner (Fig 3G), whereas growth/viability of human fibroblast host cells is not compromised (Fig 3G; insert) as expected [91–93]. Although it is well-established that mitochondrial respiration in *T*. *cruzi* is sensitive to the mammalian complex III inhibitor, antimycin A [75] this is the first demonstration of sensitivity of a kinetoplastid protozoan to endochin-like quinolones. These data agree with the recent demonstration that electron transport is a targetable process in intracellular *T*. *cruzi* [94] and support the concept that mitochondrial respiratory chain activity may be essential for *T*. *cruzi* amastigote proliferation in mammalian cells.

As an obligate intracellular *T*. *cruzi* life cycle stage, amastigotes must tap into the nutritional resources of their mammalian host cells in order to survive. The ability of *T*. *cruzi* to colonize a wide variety of mammalian cell types suggests a high degree of metabolic flexibility and the capacity for rapid adaptation. With the exception of the essential nutrients that *T*. *cruzi* is incapable of synthesizing (eg. purines, pterins) we have little knowledge of what cytosolically-localized *T*. *cruzi* amastigotes extract from their host cells or how energy metabolism is balanced in this parasite life cycle stage. While isotopic tracer experiments are required to make any definitive statements regarding nutrient uptake and utilization by intracellular *T*. *cruzi* amastigotes, the dynamic changes observed for core metabolic processes at the transcriptome level is indicative of metabolic remodeling during *T*. *cruzi* amastigote development. Similar metabolic retooling has been described in related kinetoplastid protozoan parasites [95–99]. Going forward, it will be critical to understand how *T*. *cruzi* amastigote metabolism is wired, how it is couples to host metabolic pathways, the degree of flexibility that exists within these connections and how this can change in the context of the different cell types that *T*. *cruzi* colonizes in the human host.

### Host cell response to *T*. *cruzi* infection

Transcriptomic changes induced in mammalian host cells by *T*. *cruzi* have been reported in a variety of host cell types and under different experimental conditions [100–110]. Because *T*. *cruzi* is capable of infecting most nucleated mammalian cell types, there has been little consistency among these experiments, complicating direct comparison of host transcriptional response data. Here we opted to use human foreskin fibroblasts (HFF) as the model host cell type for *T*. *cruzi* infection to facilitate comparisons to microarray hybridization studies previously conducted by our group [100, 107]. As outlined in the Methods section, our experimental approach permitted the capture of both parasite and host transcriptome response information across an infection time course *in vitro* (Fig 1A). RNA-Seq libraries generated in parallel for mock-infected HFF cultures provided the appropriate controls for each infection time point. As anticipated, some of the previously documented features of the global host transcriptional response to *T*. *cruzi* infection [100, 107] were recapitulated in the present analysis (S7 Table) as discussed below. One notable difference, however, relates to the detection of ~450 differentially expressed genes in *T*. *cruzi*-infected fibroblasts within the first 4 hpi of infection (S4 Table) contrasting sharply with the minimal response previously observed at early parasite infection time points [100]. The enhanced detection capability is likely due to the increased dynamic range and sensitivity achieved with the deep sequencing approach used here.

**Fig 4:**
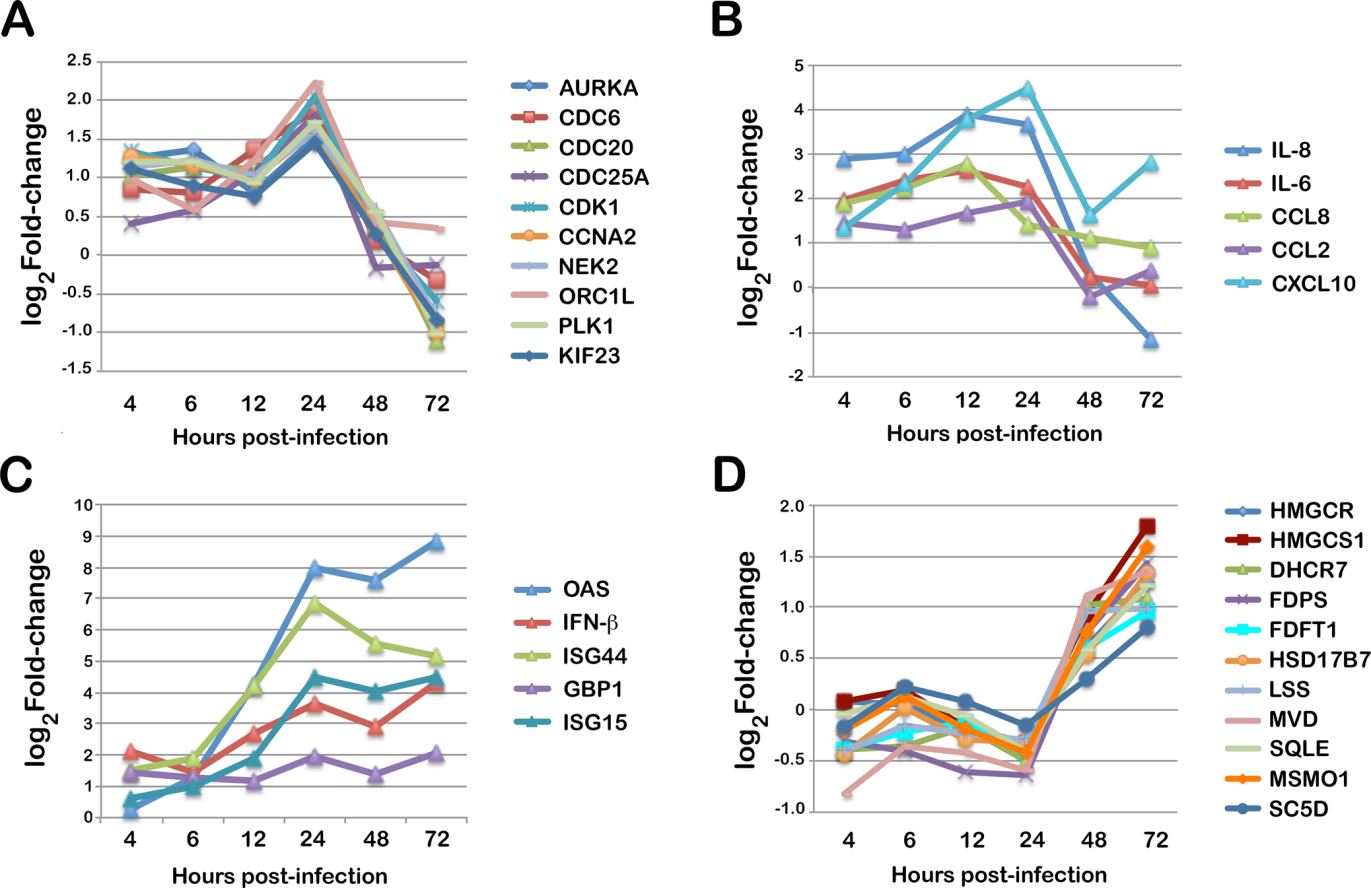
Dynamic host response signatures in *T*. *cruzi*-infected human fibroblasts. Expression patterns for selected genes in the most strongly modulated pathways in *T*. *cruzi* infected HFF. Genes in the following categories are highlighted. **(C) Mitotic Cell cycle:** *AURKA* (ENSG00000087586); *CDC6* (ENSG00000094804); *CDC20* (ENSG00000117399); *CDC25A* (ENSG00000164045); *CDK1* (ENSG00000170312); *CCNA2* (ENSG00000145386); *NEK2* (ENSG00000117650); *ORC1L* (ENSG00000085840); *PLK1* ENSG00000137807); KIF23 (ENSG00000137807). **(B) Cytokines/Chemokines:** *IL-8* (ENSG00000169429); *IL-6* (ENSG00000136244); *CCL8* (ENSG00000108700); *CCL2* (ENSG00000108691); *CXCL10* (ENSG00000169245). **(C) Type I Interferon:** *OAS* (ENSG00000089127); *IFNB* (ENSG00000171855); *ISG44* (ENSG00000137959); *GBP1* (ENSG00000117228); *ISG15* (ENSG00000187608). **(D) Mevalonate/Sterol biosynthesis:** *HMGCR* (ENSG00000113161); *HMGCS1* (ENSG00000112972); *DHCR7* (ENSG00000172893); *FDPS* (ENSG00000160752); *FDFT1* (ENSG00000079459); *HSD17B7* (ENSG00000132196); *LSS* (ENSG00000160285); *MVD* (ENSG00000167508); *SQLE* (ENSG00000104549); *MSMO1* (ENSG00000052802); *SC5D* (ENSG00000109929). All values are reported as log2 fold-change of the difference in expression of infected and matched uninfected controls at each time point as listed in S7 Table.

The Gene Ontology enrichment categories associated with the early transcriptome response in *T*. *cruzi* infected fibroblasts (4–6 hpi), while numerous (S10 Table), can be distilled into two main categories: host cell cycle progression and immune response. Among the 288 fibroblast genes that are upregulated ≥2-fold following parasite infection at 4 hpi (S4 and S7 Tables) a significant enrichment in functions related to cell cycle progression, mitosis and cell division are observed eg. GO:0000278 (S10 Table; upregulated). Plotting the mRNA expression dynamics for several cell cycle regulators (Fig 4A) shows this trend continuing until 24 hpi, after which the expression of host cell cycle genes declines precipitously (Fig 4A, S8B Fig cluster 2, and S12 Table) such that ‘mitotic cell cycle’ becomes an enriched biological process associated with downregulated host genes (S10 Table; downregulated). Overall, these observations coincide with our previous finding that *T*. *cruzi* infection pushes host cells toward S-phase in the first 24 hr of the infection cycle, with a subsequent block imposed on host cell cytokinesis at later time points [107].

An innate immune response to *T*. *cruzi* infection was also evident in the early transcriptome signature of infected fibroblasts (S10 Table; upregulated; e.g. GO:0002376) with the upregulation of pro-inflammatory cytokine and chemokine genes (Fig 4B and S7 Table) as well as type I interferon inducible genes (Fig 4C and S7 Table) with different dynamics (Fig 4C). Cytokine/chemokine gene expression peaks at 24 hpi (Fig 4B) whereas the type I IFN response (ie. genes that are expressed downstream of type I IFN receptor activation) increases gradually over the infection time course (Fig 4C) to become the dominant host transcriptomic signature by 72 hpi (S10 Table; upregulated). Differences in the expression profiles of these distinct immune response pathways is presumably related to differences in the mechanism of pathway activation by *T*. *cruzi* [111–116] and regulatory processes related to signal amplification [107]. Pro-inflammatory cytokine activation via Toll-like receptor (TLR) and cell-intrinsic response pathways is required for host protection against *T*. *cruzi* [111, 113–116]. In contrast, the type I IFN response does not require TLRs for activation in response to *T*. *cruzi* [112] and is associated with exacerbation of *T*. *cruzi* infection under instances of high parasite load to the detriment of the host [117], similar to the impact of type I IFNs on the host in a number of other non-viral pathogen infection models (reviewed in [118]). Although not required for host protection, type I IFN (*IFNA6*) and several IFN-inducible genes (*IFI44L*, *STAT1* and *GBP1*) emerged in an unbiased RNAi screen conducted in HeLa cells [119] as positively effecting the *T*. *cruzi* infection process. This finding raises the unexplored possibility that the host type I IFN response triggered by *T*. *cruzi* may be beneficial to the parasite under certain circumstances.

Corresponding with increasing intracellular parasite burden is the elevated expression of host genes related to metabolism. Included in this list are several classes of solute transporter (S7 Table) and enzymes involved in lipid biosynthesis (S8B Fig; cluster 4). In fact, most of the genes in the mevalonate pathway are upregulated ≥2-fold between 24–72 hpi (Fig 4D) as is *SREBP2* (ENSG00000198911), an important regulator cholesterol homeostasis in mammalian cells [120]. It is tempting to speculate that elevated sterol biosynthesis fuels membrane synthesis in infected host cells to accommodate a steadily increasing intracellular parasite load and/or provides a pool of sterol intermediates to be scavenged by intracellular amastigotes. However, given that cholesterol biosynthesis is an intensely oxygen-consuming process [121], it is possible that the main function of this late host response to *T*. *cruzi* is defense against oxidative stress. While the presence of replicating parasites in the host cell cytoplasm is expected to dramatically impact energy homeostasis in the host cell, energy metabolism does not emerge as an enriched GO function at any time in the intracellular infection cycle (S12 Table). Thus, the cellular response to such perturbations is predicted to occur at the post-transcriptional and post-translational levels.

The ability to simultaneously capture host cell and parasite transcriptomes with high resolution and sensitivity sets the stage for the generation of host-parasite interaction networks. The integration of transcriptome data with other types of expression data and with functional information will be instrumental in modeling the critical aspects of the parasite-host interaction and aid in the identification of targetable processes therein. As a limited exercise, we performed an intersection of datasets containing host genes that are upregulated in response to *T*. *cruzi* infection (24 hpi; S7 Table) with those previously shown to impact *T*. *cruzi* growth in a genome-scale RNAi screen [119]. Within this subset of genes (S13 Table) is *GCH1*, which encodes GTP-cyclohydrolase 1, the rate-limiting enzyme in tetrahydrobiopterin (BH_4_) synthesis. In previous work we demonstrated that siRNA-mediated knockdown of *GCH1* expression in host cells impaired intracellular *T*. *cruzi* amastigote growth in a manner that was rescued by the addition of dihydrobiopterin [119]. Here we find that *GCH1* (ENSG00000131979) is upregulated in *T*. *cruzi*-infected fibroblasts (S7 Table; ≥12 hpi) at the same time that its negative regulator (GCHI feedback regulator; ENSG00000137880) is repressed (S7 Table). Additionally, the *T*. *cruzi* gene encoding pterin-4-alpha-carbinolamine dehydratase (PCDB1: TcCLB.503613.40), an enzyme involved in biopterin recycling, was also found to be rapidly upregulated in developing intracellular amastigotes (S5 Table; 4 hpi). Together, these observations are consistent with a predicted need to increase flux through the host BH_4_ synthesis pathway to fuel the growth of intracellular *T*. *cruzi*, a pterin auxotroph [122]. With this example, it is possible to see how threads of a functional host-parasite network can emerge from data integration, an important goal of host-pathogen transcriptomic studies going forward.

### Conclusion

A key feature of our work is the demonstrated ability to parse out human and parasite sequence reads from complex pools generated from *T*. *cruzi*-infected cells and to obtain high coverage of both the parasite and host transcriptomes to enable downstream analyses with high statistical confidence. As emphasized here, the simultaneous capture of dynamic changes in host and parasite gene expression over an infection time course provides immediate and new insights into the biology of *T*. *cruzi* infection and serves as a unique resource for the construction of high-resolution maps of parasite-host interactions. Moreover, the transcriptome dynamics observed during a major life stage transition in *T*. *cruzi* revealed marked similarities between this parasite and its trypanosomatid relatives with respect to the ordering of processes that control global gene expression during differentiation. Specifically, our data support previous observations in *T*. *brucei* [12, 48] and *Leishmania donovani* [11, 13, 23, 123] indicating that regulation of mRNA levels exerts the greatest impact during the initial phases of a developmental transition in these parasites, whereas downstream mechanisms such as translational efficiency and post-translational modification dominate in the subsequent phases of development and maintenance. While regulation of gene expression in *T*. *cruzi* is understood to be multi-layered and complex (eg. [124, 125]), with translational efficiency playing a key regulatory role [18], our findings argue for the value of transcriptome data to derive meaningful biological inferences related to parasite biology. Extrapolation of this finding to the many hypothetical genes encoded in the *T*. *cruzi* genome, for which we now have dynamic mRNA expression data, has exciting implications for biological discovery in the trypanosome field. Finally, integration of transcriptome information, with emerging proteomic (eg. [87, 126–133]), functional [119] and metabolic data will, in the near future, create novel opportunities to pinpoint critical processes that govern successful pathogen colonization in the host with links to Chagas disease pathogenesis and increase the potential to identify novel targets for this important neglected disease.

## Methods

### Parasite maintenance and mammalian cell infection

*Trypanosoma cruzi* Y strain [134] was cultivated by weekly passage in LLcMK_2_ cells (ATCC #CCL-7) in Dulbecco’s modified Eagle medium (DMEM) with 2% fetal bovine serum (FBS), 2 mM L-glutamine, 10 mM HEPES and penicillin-streptomycin maintained at 37°C and 5% CO_2_. *T*. *cruzi* trypomastigotes released into the supernatants of infected LLcMK_2_ cells were collected, pelleted by centrifugation (1000*g*, 10 min) and collected from the supernatant after swimming up from the pellet over a 2–4 hour incubation at 37°C, 5% CO_2_. Human foreskin fibroblasts (HFF) (ATCC #CRL-2522) were seeded onto 10 cm^2^ plates or T-25 flasks in complete DMEM (as above, with 10% FBS) and grown to 80% confluence (48 hr) before infection. Briefly, HFF monolayers were washed with DMEM-2%FBS (DMEM-2) and either incubated with medium (mock) or *T*. *cruzi* trypomastigotes for 2h before washing 5 times with PBS and incubation in DMEM-2 at 37°C and 5% CO_2_. At the indicated time points (4–72 hpi), cells were rinsed with ice-cold PBS and lysed directly in Trizol for RNA isolation. *T*. *cruzi* epimastigotes, maintained at mid-log phase in liver infusion tryptose medium at 27°C, or purified extracellular trypomastigotes (>95% pure) were also used for RNA isolation. To obtain intracellular *T*. *cruzi* amastigotes for metabolic studies, infected LLcMK_2_ (60 hpi) were washed with ice-cold Krebs-Henseleit Buffer (KHB) containing 0.5mM glucose, and scraped into 1ml ice-cold KHB + 0.5mM glucose with a cell scraper. Dislodged cells were collected in 9ml final volume of KHB + 0.5mM glucose in a 50ml tube, and centrifuged at 2100g for 10 minutes at 4°C. Pelleted cells were resuspended in 1ml of KHB + 0.5mM glucose, transferred to Eppendorf tubes, vortexed for 45s and passed through a 1ml syringe with a 28.5G needle 20 times to release amastigotes. Unbroken cells and debris were pelleted at 100g for 1 minute and the amastigote-enriched supernatant was pelleted at 1000g for 10 minutes at room temperature. Amastigotes were resuspended in warm (37°C) KHB + 0.5mM glucose at 2x10^7^ amastigotes per ml.

### RNA isolation and library construction for simultaneous transcriptome profiling of parasite and host cells

RNA was isolated in Trizol reagent, as per manufacturer, and the quality determined using an Agilent 2100 bioanalyzer and quantified by qPCR using a KAPA Biosystems library quantification kit. Standard Illumina protocols were used for mRNA-Seq sample preparation. RNA-Seq libraries were constructed from polyA-enriched mRNAs generated from eight *T*. *cruzi* developmental stages: epimastigotes, trypomastigotes, and intracellular amastigotes at 4, 6, 12, 24, 48 and 72 hrs post-infection (hpi) of HFF. Libraries were also constructed from mock-infected HFF at the same time points. For each condition, 2–4 independent biological replicates were sequenced on an Illumina HiSeq1000. A total of 2.7 billion reads from 35 samples were generated from 101 bp paired-ends.

### Sequence read processing, alignment, abundance estimation and data normalization

The quality of the raw reads was evaluated using the FastQC tool [http://www.bioinformatics.babraham.ac.uk/projects/fastqc/] and one nucleotide was trimmed using the FASTX toolkit (Hannon Lab, CSHL) when the mean of the quality score fell below 30 in the last position (see analysis pipeline S1 Table). Tophat v2.0.8 [36] was used to align all reads to the reference human genome sequence (hg19, GRCh37) and independently to the of the *T*. *cruzi* CL Brener reference genome (v. 4.1) [35] Esmeraldo haplotype obtained from the TriTrypDB database (www.tritrypdb.org). Alignment settings allowed 2 mismatches per read and the default -g/—max-multihits parameter of -g = 20 was used for alignments to the human genome. A parameter of -g = 1 was used for *T*. *cruzi* to allow reads to map to a single locus in this organism where multi-gene families are abundant. The read counts per coding sequence (CDS) were determined using HTSeq [http://www-huber.embl.de/users/anders/HTSeq/] as listed *T*. *cruzi* (S2 Table) and human (S3 Table) genes.

### Data quality assessment by statistical sampling and visualization

Weakly expressed genes, defined as having less than 1 read per million in ‘n’ of the samples, where ‘n’ is the size of the smallest group of replicates [135] (here n = 2 and 3 for the *T*. *cruzi* and human samples, respectively) were removed from subsequent analyses. Pearson correlation and standardized median correlation analyses, box plots, Principal Component Analysis (PCA) and Euclidean distances-based hierarchical clustering approaches were used to evaluate replicates and the relationships between samples across time points and to visualize sample-sample distances. All components of our statistical pipeline, named cbcbSEQ, can be accessed on GitHub (https://github.com/kokrah/cbcbSEQ/). Samples that did not pass the following quality assessment procedure were removed: for each sample we computed the median pairwise correlation (mpc) to all other samples in the dataset (S5 Fig). A standard outlier identification method was then applied to remove samples with low correlation to the other samples: samples were removed if their median pairwise correlation (mpc) is less than Q1(mpc)– 1.5 IQR(mpc) where Q1(mpc) and IQR(mpc) are the first quartile and inter-quartile range of the median pairwise correlation across all samples respectively. HPGL0111 was removed from subsequent analyses as a result.

### Differential expression analysis

A quantile normalization scheme was applied to all samples [86]. Following log2 transformation of the data, Limma [136] was employed for differential expression analyses. Limma utilizes a standard variance moderated across all genes using a Bayesian model and produces *p*-values with greater degrees of freedom [137]. When appropriate, the Voom module was used to transform the data based on observational level weights derived from the mean-variance relationship. Experimental batch effects were adjusted by including experimental batch as a covariate in our statistical model. Type I error introduced by multiple testing was corrected with q-value [138]. A contrast matrix was used within Limma. To control for expression profile changes in human cells that occur naturally over time in cell culture, normalized-log2-transformed expression values for each gene in ‘uninfected’ was subtracted from ‘infected’ in the paired uninfected/infected HFF samples at each time point. Differentially expressed genes were defined as genes with q-value < 0.05.

### Filtering *T*. *cruzi* DEG lists to remove paralogous genes

Each list of differentially expressed *T*. *cruzi* genes generated from pairwise comparisons (S5 Table) was submitted to a search against itself using FASTA36 [139]. Groups of genes were counted as paralogous when observed with an e-value ≤0.0001 and percentage identity ≥80%. The first lexically listed gene in each group was taken as a representative for the group shown in S6 Table. It should be noted that while a large proportion of paralogous genes have been collapsed, a number of truncated (partial) genes as well as genes encoding large hypervariable regions (i.e. MASP) can only be manually removed, a process more prone to error due to reliance on manual curation.

### K-means clustering and functional enrichment analysis

K-means clustering analysis was performed to identify genes with similar expression profiles across different developmental stages of *T*. *cruzi* or human host cells with the R function “kmeans” and using the Hartigan-Wong algorithm. Quantile-normalized and batch effect-adjusted expression values were used for clustering and Euclidean distance was computed as the distance metric; 8 partitions were used to generate the clusters following the method of [41, 140]. Lists of significantly regulated genes resulting from differential expression or clustering analyses were classified into GO functional categories and tested for enrichment using GOSeq, which applies Wallenius approximation to correct the bias of over-detection of differential expression for long and highly expressed transcripts [40]. False discovery rate (FDR) was controlled using the Benjamini and Hochberg's procedure [141].

### Metabolic analyses

Mitochondrial respiratory capacity was measured using an XF^e^24 extracellular flux analyzer (Seahorse Biosciences). Briefly, XF^e^24 assay plates were pre-coated with 30 μl of 7.7% Cell-Tak (Corning) in 100 mM sodium bicarbonate, pH 8 for 30 minutes, then the wells were washed three times with 0.5 ml warm Krebs-Henseleit Buffer (KHB) before plating parasites. Isolated *T*. *cruzi* (Tulahuen strain) [142] trypomastigotes and amastigotes were resuspended in either XF Base Medium (Seahorse Biosciences) + 10mM glucose, 2mM glutamine, and 1mM sodium pyruvate or KHB + 0.5mM glucose at 2x10^7^ parasites/ml. 2x10^6^ parasites in 100 μl were delivered to each well of a Cell-Tak pre-coated Seahorse XF^e^24 assay plate and immediately centrifuged at 2056g for 2 minutes. The volume of medium in each well was adjusted to a total volume of 450 μl/well of plating medium. To determine basal respiratory capacity, ATP-coupled respiration, and spare respiratory capacity, drugs from the XF Cell Mito Stress Test Kit (Seahorse Biosciences) were resuspended in warm media and injected at 10x their final well concentrations of 2.5μM oligomycin, 3μM carbonyl cyanide-4-(trifluoromethoxy)phenylhydrazone (FCCP), and a mixture of 1μM antimycin A and rotenone, in order. Results were normalized to parasite DNA based on a quantitative PCR assay of the single copy *T*. *cruzi* gene: OSBP (TcCLB.508211.10). At the end of the run, the media was replaced with 200 μl PBS and the plate was frozen at -20°C. DNA was isolated after thawing the plate using the DNeasy Blood and Tissue Kit (Qiagen). For qPCR, 10 μl of iTaq Universal SYBR Green Supermix (BioRad) was combined with 0.33 μl isolated DNA, 1 pmol each of primers to amplify a single copy *T*. *cruzi* gene OSBP (F: 5’-CAT CAC CTA CGG CCA CAA GA-3’, R: 5’-TGC AGT GGA TAC GCA TAC GG-3’), and water for a 20 μl reaction volume. The reaction was run at 95°C for 5 minutes, then cycled 45 times at 95°C for 15 seconds followed by 60°C for 60 seconds. The amount of DNA per Seahorse plate well was calculated by comparing Ct values to a standard curve generated by 1:2 dilutions of 10 ng of *T*. *cruzi* DNA. To calculate basal respiratory capacity, the normalized oxygen consumption rates (OCR) after antimycin A and rotenone addition was subtracted from the baseline OCR. For ATP-coupled respiration, normalized OCR after oligomycin injection was subtracted from baseline OCR. Spare respiratory capacity was calculated as the difference between normalized OCR after FCCP injection and control wells at the same timepoint without any drug injections. Glutamine (Gibco, Life Technologies) and ELQ271 (generously supplied by M. Riscoe; OHSU) were resuspended in warm medium and injected at 10x their final well concentrations of 2mM and 10μM, respectively.

### Total ATP determination

To measure total ATP levels, *T*. *cruzi* trypomastigotes and amastigotes were isolated (as above) and resuspended in Krebs-Henseleit Buffer (KHB) at 4x10^6^ parasites/ml. Trypomastigotes and amastigotes were plated at 4x10^5^ parasites in 100μl in a separate white 96-well plates (Corning) for each time point and incubated at 37°C. Parasites were lysed and ATP levels were determined using the ATPlite assay kit (PerkinElmer), measuring luminescence on a Varioskan Flash plate reader (ThermoScientific).

### Drug susceptibility assay

HFF were seeded at 1500 cells/well in 384 well black bottom plates (Corning). At 24h post plating, cells were infected with β-galactosidase expressing *T*. *cruzi* Tulahuen strain (moi 5) for 2hr, washed twice, and left in DMEM (2% FCS, 2 mM glutamine, 1 mM pyruvate). At 18hpi cells were treated with 0.3–10 μM of ELQ271. At 72 hpi HFF viability was measured in a fluorescence-based readout (CellTiter-Fluor, Promega) and *T*. *cruzi* was measured by luminescence-based readout (Beta-Glo reagent, Promega) using an Envision Plate Reader (PerkinElmer) as described [32]. The relative infection (RLU/RFU) was calculated and normalized to untreated control fitted by non-linear regression to high (= 100%) and low (= 0%) values using GraphPad Prism software.

### Quantitative RT-PCR

RNA was isolated from purified *T*. *cruzi* trypomastigotes and from infected monolayers (at the indicated timepoints post infection) following cell lysis in Trizol reagent and purification using the PureLink RNA Mini Kit (Ambion). DNase-digested (Turbo DNase, Ambion) total RNA (1 μg) was converted to cDNA using the iScript (Bio-Rad) cDNA synthesis kit according to manufacturer’s instructions. Specific primer pairs to amplify genes of interest in quantitative-RT-PCR reactions were selected on their ability to form single peak in melting curve analysis and verified by sequencing of PCR products. Forward (F) and reverse (R) primer pairs for are listed below in a 5’ to 3’ orientation. Cation transporter (TriTrypDB: TcCLB.509197.39) F: GAGTGTCATGCTTGAAGTG and R: CGTTAAAAATAAGAGAAAATG; Glutamate dehydrogenase (TriTrypDB: TcCLB.507875.20) F: GAGTACTGCCAGGATTCTC and R: CAAAGCCAAGAAACTTAAG; Fatty acid elongase (TriTrypDB: TcCLB.506661.30) F: GAGGCAACCTGCACATTTAAC and R: GTGTCCATCAACTCAGGAATCT; Fatty acid desaturase (TriTrypDB: TcCLB.511073.10) F: AAGGAACGTGAAGAATCTC and R: AACGGACTTCTCCAGATC; Hypothetical protein, conserved (TriTrypDB: TcCLB.509767.170) F: ATGAAGCTTGCGTTCTCT and R: GGTCACAATAGCCCAGTC; Ribosomal RNA large subunit gamma M1 (TriTrypDB: TcCLB.411483.20) F: TGTGGAAATGCGAAACAC and R: CCCAGGTTTTTGCTTTAATG. Relative mRNA transcript abundance was quantified by SYBR green (iTaq Universal SYBR Green Supermix, Bio-Rad) PCR using a StepOnePlus Real-Time PCR Systems (Applied Biosystems). Large subunit ribosomal RNA gamma (M1): TcCLB.411483.20) used as endogenous control.

### Data accession

RNA-Seq data are available at the National Center for Biotechnology (NCBI) Sequence Read Archive (SRA) (http://www.ncbi.nlm.nih.gov/bioproject) under Bioproject PRJNA251582 (accession numbers ranging from SRR1346026-SRR1346052) and Bioproject PRJNA251583 (accession numbers ranging from SRR1346053-SRR1346059). Individual accession numbers are also shown in S1 Table.

## Supporting Information

S1 Fig Schematic of pipeline for differential expression analysis. The input/output for each step of the data processing and analysis is depicted in rectangular boxes. Software/scripts or methodological components are shown in italicized blue text. Decision-making steps are represented diamond-shaped boxes. The five stages to the analysis are shown in the left margin. The main figures or supplemental files in which data outputs from key steps in the pipeline can be found are highlighted in red.
(PDF)

S2 Fig Distribution of global gene expression levels in all *T*. *cruzi* and human samples. For all samples from *T*. *cruzi* (**A**) intracellular and (**B**) extracellular stages, and (**C**) human, counts were normalized for sequencing library size and a box plot was generated to compare the distribution of per-gene counts (log2 counts per million with an offset of 1). The ends of the whiskers represent the lowest datum still within 1.5 interquartile range (IQR) of the lower quartile, and the highest datum still within 1.5 IQR of the upper quartile. Genes with extremely high or low expression levels are shown as open circles above and below the whiskers, respectively. Mapped read counts from all parasite and human cell samples showed consistent degrees of dispersion as indicated by the nearly identical quartile distributions in similar samples. The median expression values for *T*. *cruzi* genes display a more compact distribution than that observed for the human genes.
(PDF)

S3 Fig Heatmap of Pearson correlations. Gene counts were normalized for sequencing library size. All pairwise Pearson correlations were calculated and plotted as a heatmap to view the relatedness of samples and identify outliers for **(A)** *T*. *cruzi* and **(B)** human.
(PDF)

S4 Fig Pairwise Pearson correlation between *T*. *cruzi* samples. Gene counts were normalized for sequencing library size. The Pearson correlation between each sample and all other samples was calculated and plotted to view the relatedness of samples and identify outliers.
(PDF)

S5 Fig Pairwise Pearson correlation between human samples. Gene counts were normalized for sequencing library size. The Pearson correlation between each sample and all other samples was calculated and plotted to view the relatedness of samples and identify outliers.
(PDF)

S6 Fig Standardized median Pearson correlation between *T*. *cruzi* and human samples. Gene counts were normalized for sequencing library size. The standardized median Pearson correlation between each sample and all other samples was plotted to view the relatedness of samples and identify outliers for **(A)** intracellular *T*. *cruzi*; **(B)** extracellular *T*. *cruzi* and **(C)** human samples. Letters in the sample name refer to experimental batch.
(PDF)

S7 Fig Hierarchical clustering of *T*. *cruzi* and human samples. Hierarchical clustering analysis based on Euclidean distance was performed using all **(A)** *T*. *cruzi* or **(B)** Human genes after filtering for weakly expressed genes, quantile normalization, and inclusion of the batch variable in the statistical model used by **Limma**. Colors along the top of the heatmap indicate the developmental stage and colors along the left side of the heatmap indicate the batch/experimental date.
(PDF)

S8 Fig K-means clustering of gene expression in *T*. *cruzi* and human cells during the course of infection. K-means clustering of genes from (**A**) *T*. *cruzi* and (**B**) human across the intracellular infection course were presented. Log2-tranasformed and quantile-normalized batch-adjusted gene expression values (y-axis) are plotted across the seven conditions (trypo, 4, 6, 12, 24, 48, 72 hpi) for *T*. *cruzi* and six time points for human (4, 6, 12, 24, 48, 72 hpi) on the x-axis. Genes included in each of the clusters are listed in S11 Table and S12 Table.
(PDF)

S9 Fig Independent validation of selected developmentally regulated metabolic genes in *T*. *cruzi*. Expression of selected *T*. *cruzi* transcripts in intracellular infection stages (6–72 hr post-infection) relative to extracellular trypomastigotes (expression level arbitrarily set to 1). Data derived from RNA-Seq differential expression analysis **(A)** or qRT-PCR **(B)** is shown for the following *T*. *cruzi* (Y strain) genes: TcCLB.509197.39: Cation transporter (CAT); TcCLB.507875.20: glutamate dehydrogenase (GlutDH); TcCLB.508373.20: dihydroorotase (DHO); TcCLB.506661.30: fatty acid elongase (FAE); TcCLB.511073.10: fatty acid desaturase (FAD) and TcCLB.509767.170: hypothetical protein (HYP). Error bars in (B) represent the mean of duplicate samples.
(PDF)

S10 Fig Temporal expression of selected *T*. *cruzi* RNA-binding proteins and flagellum-associated genes. Relative mRNA levels of **(A)** *T*. *cruzi* RNA-binding proteins and **(B)** flagellar genes that were differentially expressed in at least one of the intracellular amastigote stages (4–72 hpi) as compared to extracellular trypomastigotes (**T**).
(PDF)

S1 Table Samples collected and mapping statistics. Complete description of all samples included in this analysis, including sample ID, SRA accession number, developmental stage, infection status, experimental batch, trimming information, number of raw reads, and number and percentage of reads mapped to each reference genome.
(XLSX)

S2 Table Raw mapped read counts and log-transformed quantile-normalized cpm expression values for *T*. *cruzi* genes. Tophat was used to align *T*. *cruzi* cDNA reads to align to the reference genome as described in Methods. The abundance of reads mapping to each coding sequence (CDS) was determined using HTSeq (Raw reads). Weakly expressed genes, defined as having less than 1 read per million in ‘n’ of the samples, where ‘n’ is the size of the smallest group of replicates (here n = 2) were removed from subsequent analyses. A quantile normalization scheme was applied to all samples. Following log2 transformation of the data, the count per million values (cpm) were calculated for each gene using in-house script. All components of our statistical pipeline, named cbcbSEQ, can be accessed on GitHub (https://github.com/kokrah/cbcbSEQ/).
(XLSX)

S3 Table Raw mapped read counts and log-transformed quantile-normalized cpm expression values for human genes. Tophat was used to align human cDNA reads to align to the reference genome as described in Methods. The abundance of reads mapping to each coding sequence (CDS) was determined using HTSeq (Raw reads). Weakly expressed genes, defined as having less than 1 read per million in ‘n’ of the samples, where ‘n’ is the size of the smallest group of replicates (here n = 3) were removed from subsequent analyses. A quantile normalization scheme was applied to all samples. Following log2 transformation of the data, the count per million values (cpm) were calculated for each gene using in-house script. All components of our statistical pipeline, named cbcbSEQ, can be accessed on GitHub (https://github.com/kokrah/cbcbSEQ/).
(XLSX)

S4 Table Summary of Differentially Expressed Genes (DEGs). The number of differentially expressed genes (DEG) obtained with Limma software with t-statistic for *T*. *cruzi* or human fibroblast expression data before and after imposing a ≥ 2-fold cut-off filter (abs(log_2_FC) ≥ 1.0). *T*. *cruzi* DEG lists were further filtered to collapse paralogous genes to a single representative gene as described in the Methods.
(XLSX)

S5 Table Report of differential expression analysis for *T*. *cruzi* genes. Each sheet reports differential expression results for a specific pairwise comparison. The columns include Gene ID, gene function, log2 fold change (FC), *P* value, q-value, adjusted *P* value, average expression value, qvals.fc, and adjPvals.fc. The latter two are Booleans set to 1 when abs(log_2_ FC) ≥ 1.0 and q-value or adjusted *P* value < 0.05 respectively. Genes are considered differentially expressed when the qvals.fc = 1.
(XLSX)

S6 Table *T*. *cruzi* DEGs with paralogues collapsed. Differentially expressed genes from S5 Table were filtered to remove all but a single representative of each paralogous gene group. These filtered gene lists served as the starting point for k-means clustering and functional enrichment analyses.
(XLSX)

S7 Table Report of differential expression analysis for human genes. Each sheet reports differential expression results for a specific comparison. The columns include gene id, gene function, log2 fold change, *P* value, q-value, adjusted *P* value, average expression value, qvals.fc, and adjPvals.fc. The latter two are Booleans set to 1 when abs(log_2_ FC) ≥ 1.0 and q-value or adjusted *P* value < 0.05 respectively. Genes are considered differentially expressed when the qvals.fc = 1.
(XLSX)

S8 Table Overrepresented *T*. *cruzi* genes by developmental stage. Each sheet contains a list of *T*. *cruzi* genes that are expressed at a significantly higher level (≥2-fold difference in expression) in one parasite life cycle stage as compared with the other two life stages with amastigote transcriptome data from the 24 hpi infection time point serving as the representative amastigote sample.
(XLSX)

S9 Table Enriched Gene Ontology terms in differentially expressed *T*. *cruzi* genes. The enriched Gene Ontology (GO) terms associated with the differentially expressed genes (DEG) emerging from pairwise comparisons of *T*. *cruzi* life stages (listed in S6 Table). Data are presented for each GO domain (biological process, molecular function, cellular component) in separate worksheets. GO term, total number of genes in the GO category, number of DEGs present within the category, *P* value for the relevant GO category over-represented among DEGs in the comparison, adjusted *P* value and description of the enriched GO category included.
(XLSX)

S10 Table Enriched Gene Ontology terms in differentially expressed human genes. The enriched Gene Ontology (GO) terms associated with the differentially expressed genes (DEG) emerging from pairwise comparisons of human samples (listed S7 Table) DEG lists were filtered by collapsing paralogous gene families prior to conducting GO enrichment analysis. Data are presented for each GO domain (biological process, molecular function, cellular component) in separate worksheets with GO term, total number of genes in the GO category, number of DEGs present within the category, *P* value for the relevant GO category over-represented among DEGs in the comparison, adjusted *P* value and description of the enriched GO category included.
(XLSX)

S11 Table Enriched Gene Ontology terms in *T*. *cruzi* gene clusters. Enriched Gene Ontology (GO) terms associated with K-means gene clusters of differentially expressed *T*. *cruzi* genes (shown in S8A Fig) over the course of a human fibroblast infection. Columns contain (A) Name of the K-means cluster corresponding to those in S8A Fig; (B) GO category; (C) Total number of genes in category; (D) Number of differentially expressed genes (DEG) in category; (E) *P* value for over-represented genes in category; (F) the adjusted *P* value and (G) Gene ontology term.
(XLSX)

S12 Table Enriched Gene Ontology terms in human gene clusters. Enriched Gene Ontology (GO) terms associated with K-means gene clusters of differentially expressed human fibroblast genes associated with a *T*. *cruzi* infection time course (shown in S8B Fig). Columns contain (A) Name of the K-means cluster corresponding to those in S8B Fig; (B) GO category; (C) Total number of genes in category; (D) Number of differentially expressed genes (DEG) in category; (E) *P* value for over-represented genes in category; (F) the adjusted *P* value and (G) Gene ontology term.
(XLS)

S13 Table Intersection of host cell expression and functional datasets. List of human genes arising from the intersection of two datasets: **(1)** the set of human fibroblast genes that are upregulated in *T*. *cruzi* infected cells at 24 hpi (from S7 Table; 838 genes) and **(2)** the set of primary hits from a human genome-wide siRNA screen that were identified as permissive for *T*. *cruzi* infection [119] (382 genes).
(XLSX)
