# Supplementary figures and images for: Transcriptome Remodeling in Trypanosoma cruzi and Human Cells during Intracellular Infection
Source: PLoS Pathog. 2016 Apr 5;12(4):e1005511. doi: 10.1371/journal.ppat.1005511 (PMC4821583; doi:10.1371/journal.ppat.1005511)

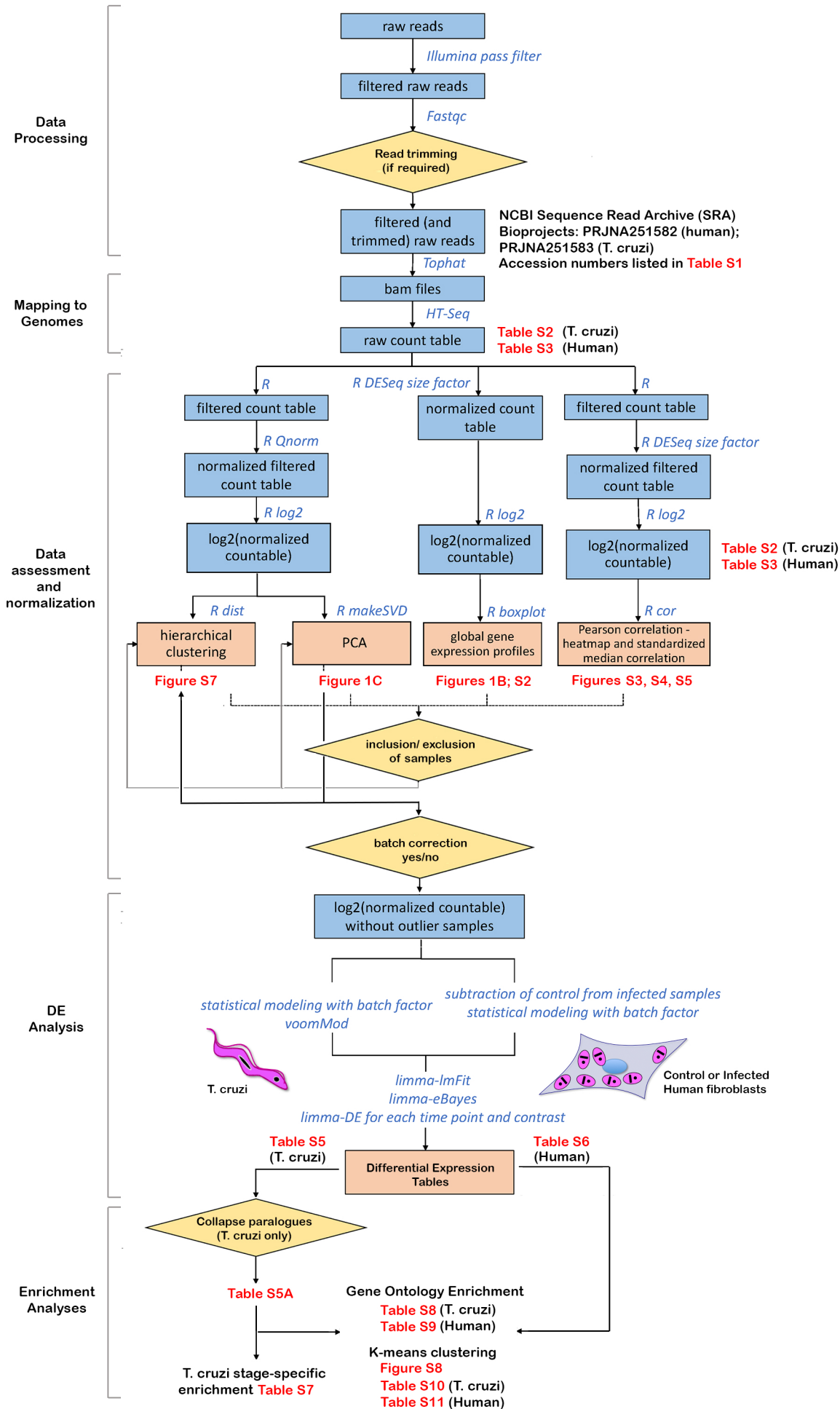

Supplement: S1 Fig — The input/output for each step of the data processing and analysis is depicted in rectangular boxes. Software/scripts or methodological components are shown in italicized blue text. Decision-making steps are represented diamond-shaped boxes. The five stages to the analysis are shown in the left margin. The main figures or supplemental files in which data outputs from key steps in the pipeline can be found are highlighted in red. (PDF) [file ppat.1005511.s001.pdf]

**A**Intracellular *T. cruzi* samples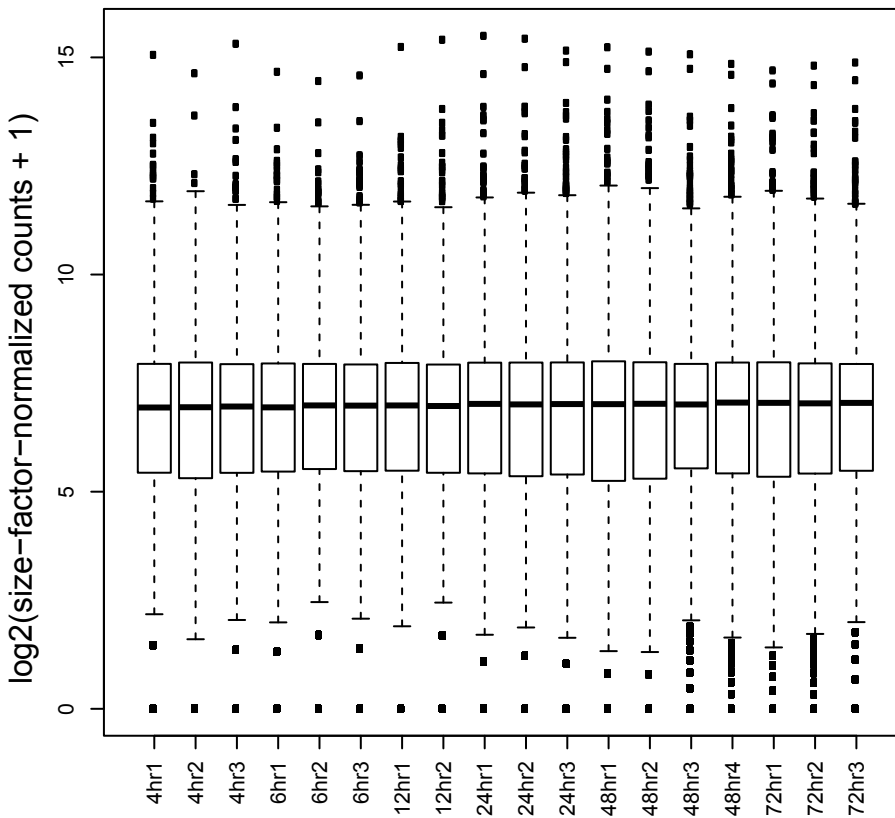**B**Extracellular *T. cruzi* samples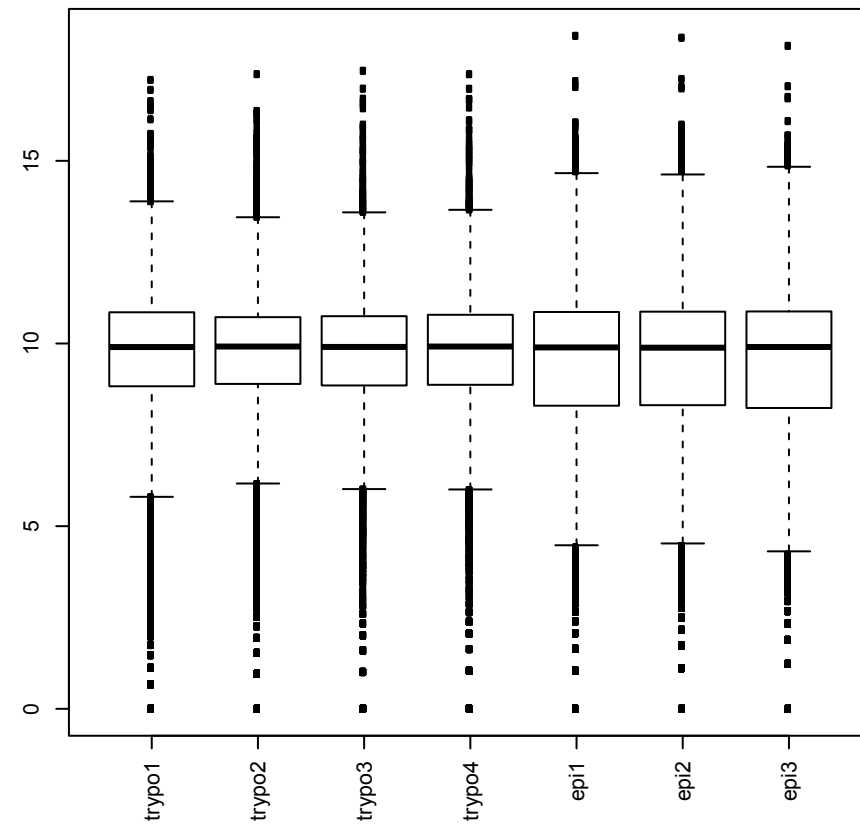**C**

All human samples

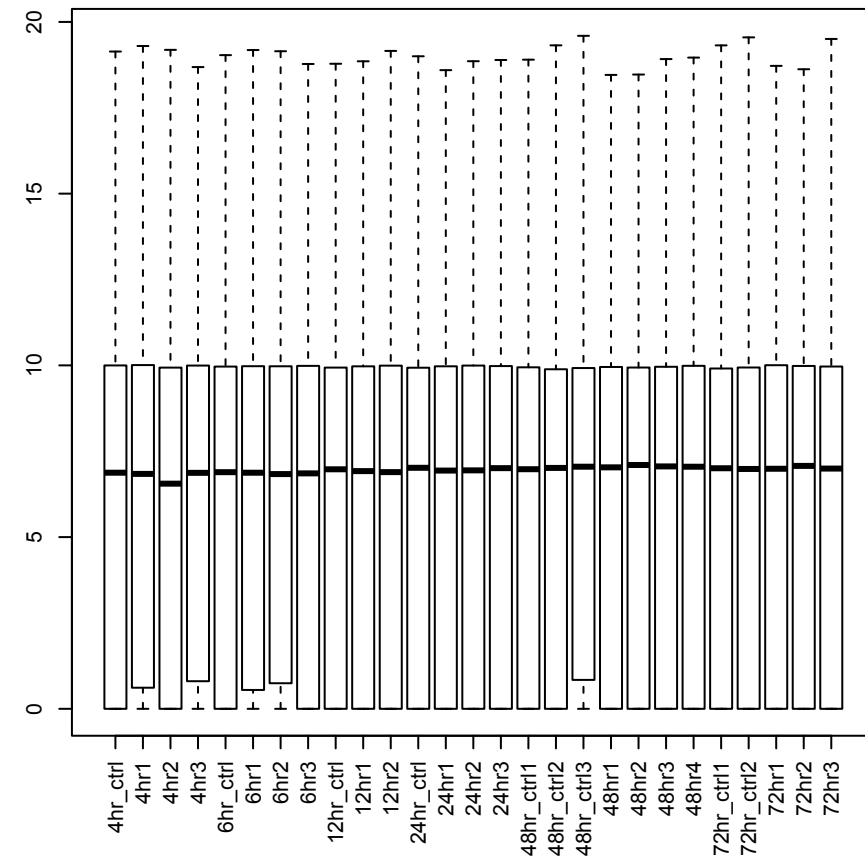

Supplement: S2 Fig — For all samples from T. cruzi (A) intracellular and (B) extracellular stages, and (C) human, counts were normalized for sequencing library size and a box plot was generated to compare the distribution of per-gene counts (log2 counts per million with an offset of 1). The ends of the whiskers represent the lowest datum still within 1.5 interquartile range (IQR) of the lower quartile, and the highest datum still within 1.5 IQR of the upper quartile. Genes with extremely high or low expression levels are shown as open circles above and below the whiskers, respectively. Mapped read counts from all parasite and human cell samples showed consistent degrees of dispersion as indicated by the nearly identical quartile distributions in similar samples. The median expression values for T. cruzi genes display a more compact distribution than that observed for the human genes. (PDF) [file ppat.1005511.s002.pdf]

A

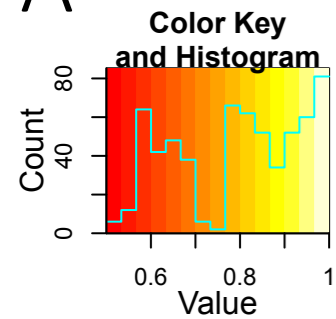*T. cruzi*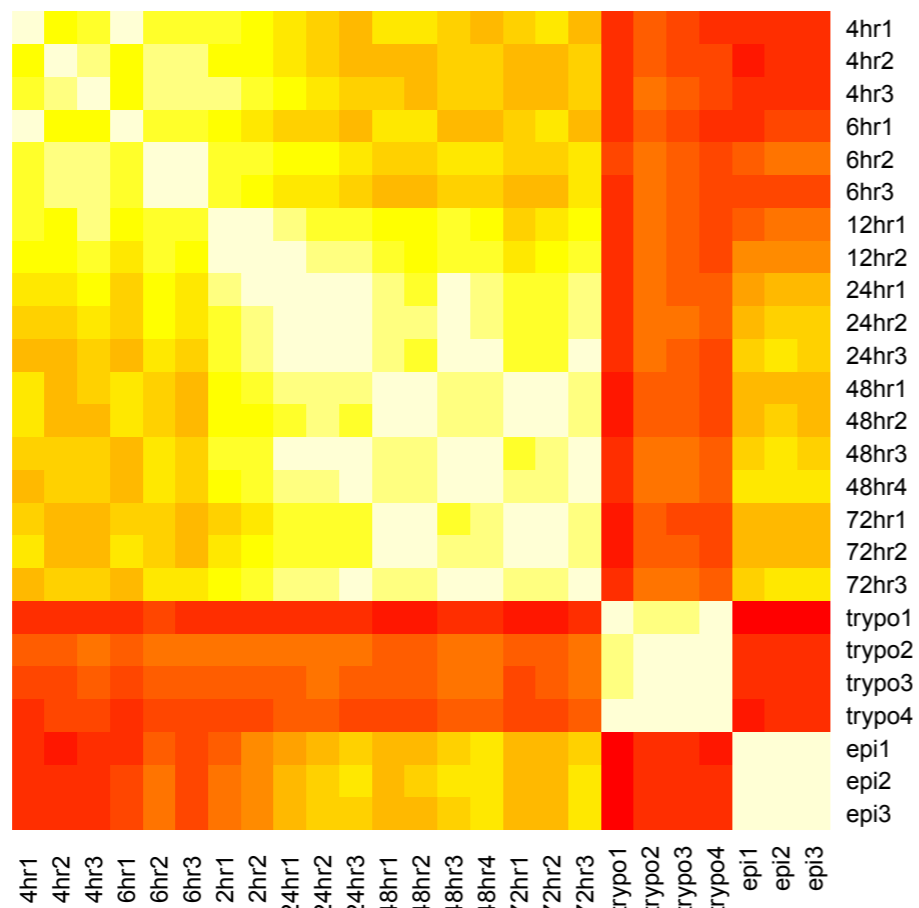

B

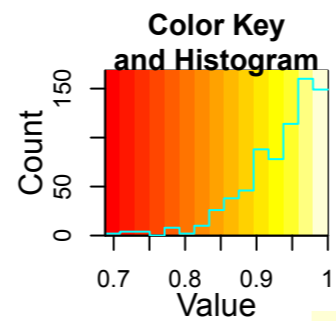

human

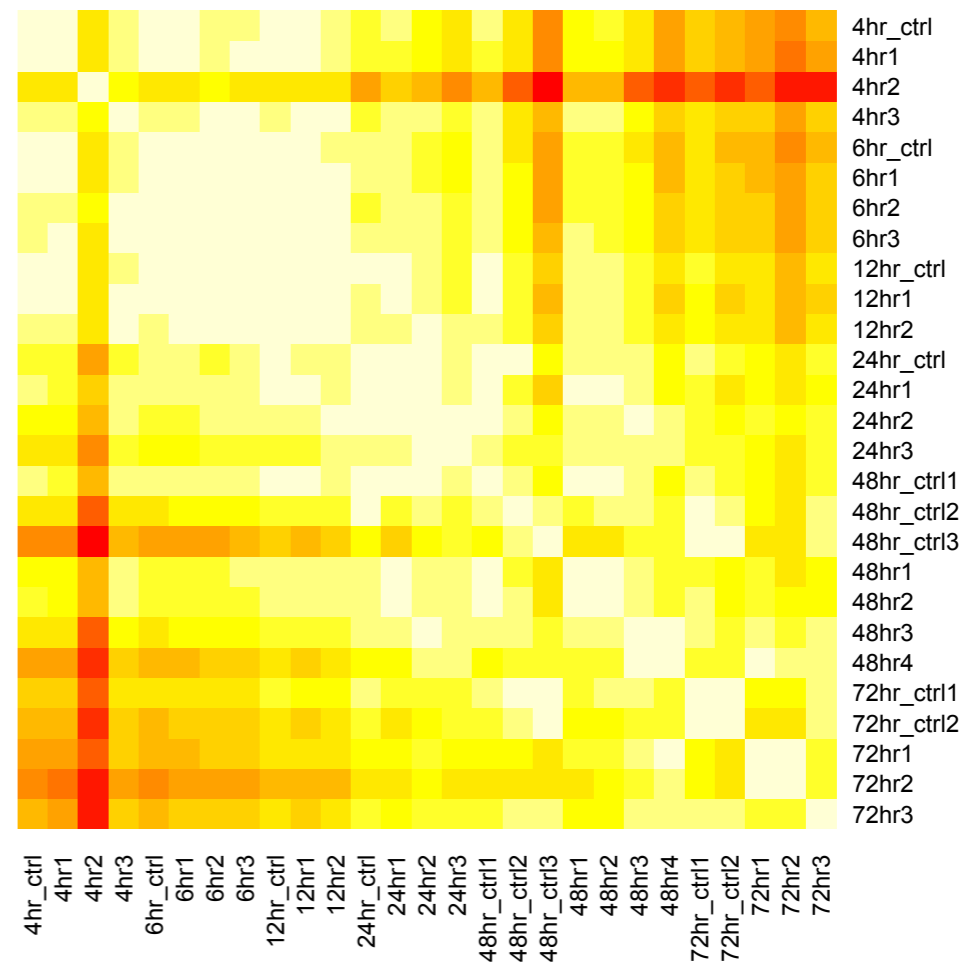

Supplement: S3 Fig — Gene counts were normalized for sequencing library size. All pairwise Pearson correlations were calculated and plotted as a heatmap to view the relatedness of samples and identify outliers for (A) T. cruzi and (B) human. (PDF) [file ppat.1005511.s003.pdf]

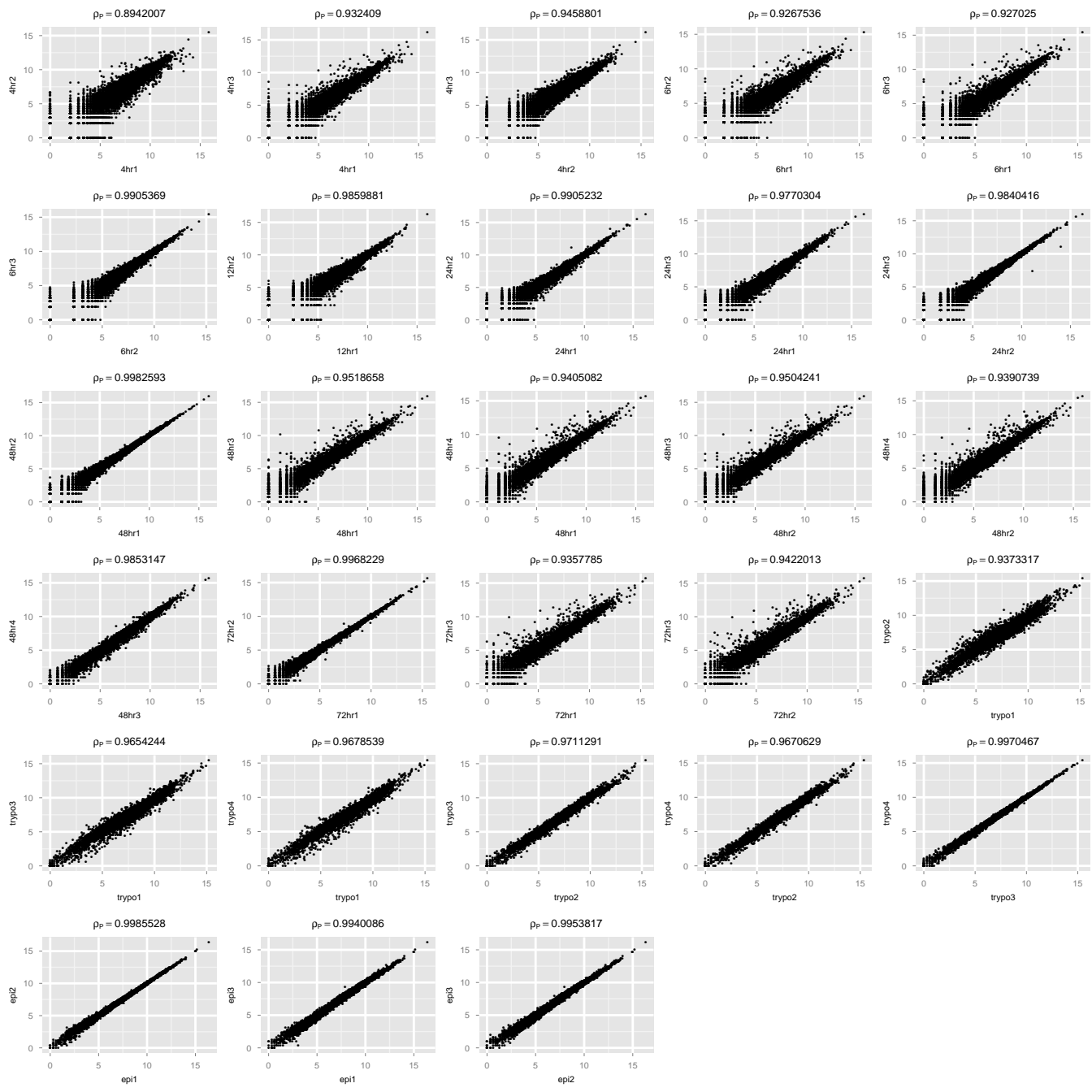

Supplement: S4 Fig — Gene counts were normalized for sequencing library size. The Pearson correlation between each sample and all other samples was calculated and plotted to view the relatedness of samples and identify outliers. (PDF) [file ppat.1005511.s004.pdf]

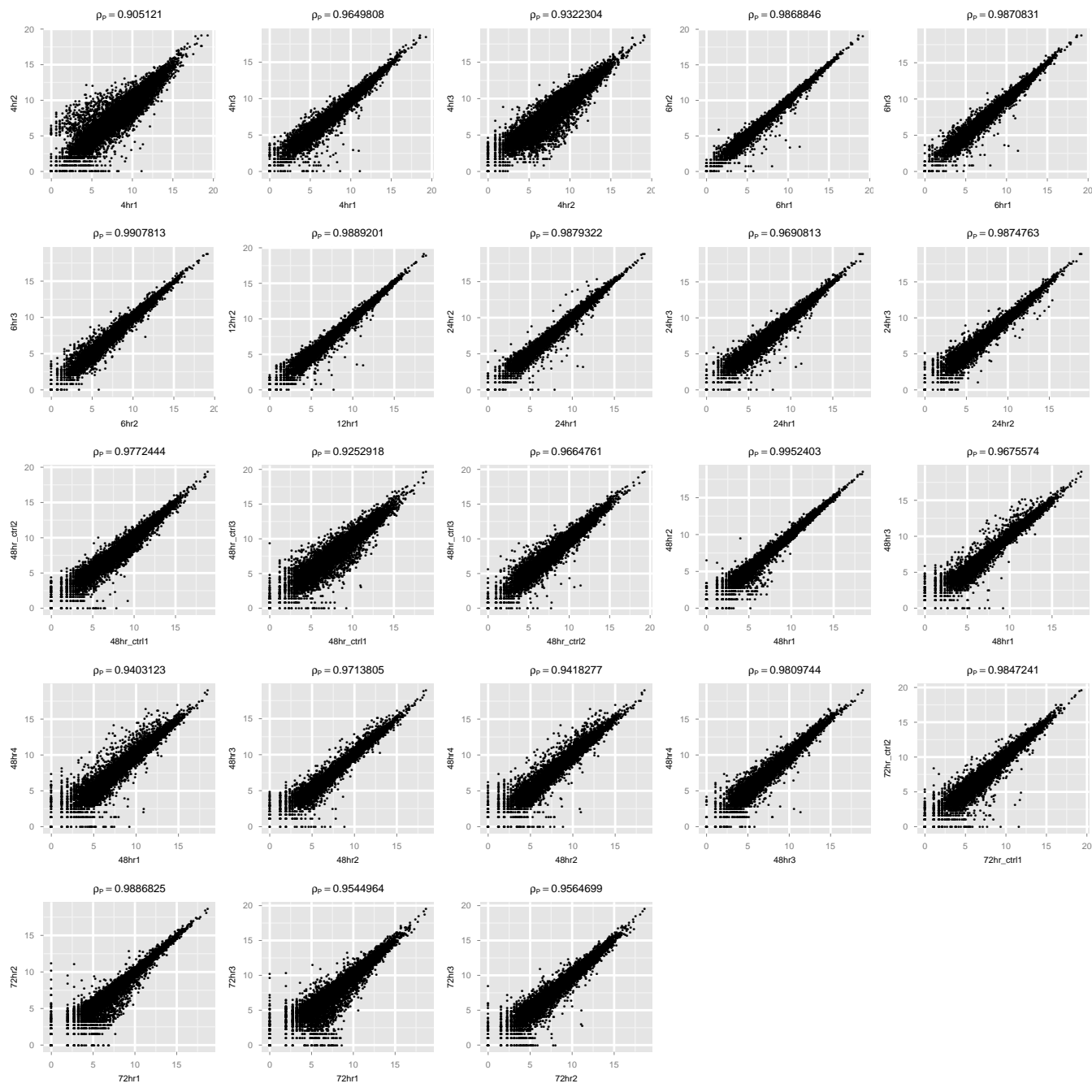

Supplement: S5 Fig — Gene counts were normalized for sequencing library size. The Pearson correlation between each sample and all other samples was calculated and plotted to view the relatedness of samples and identify outliers. (PDF) [file ppat.1005511.s005.pdf]

A

Intracellular *T.cruzi* samples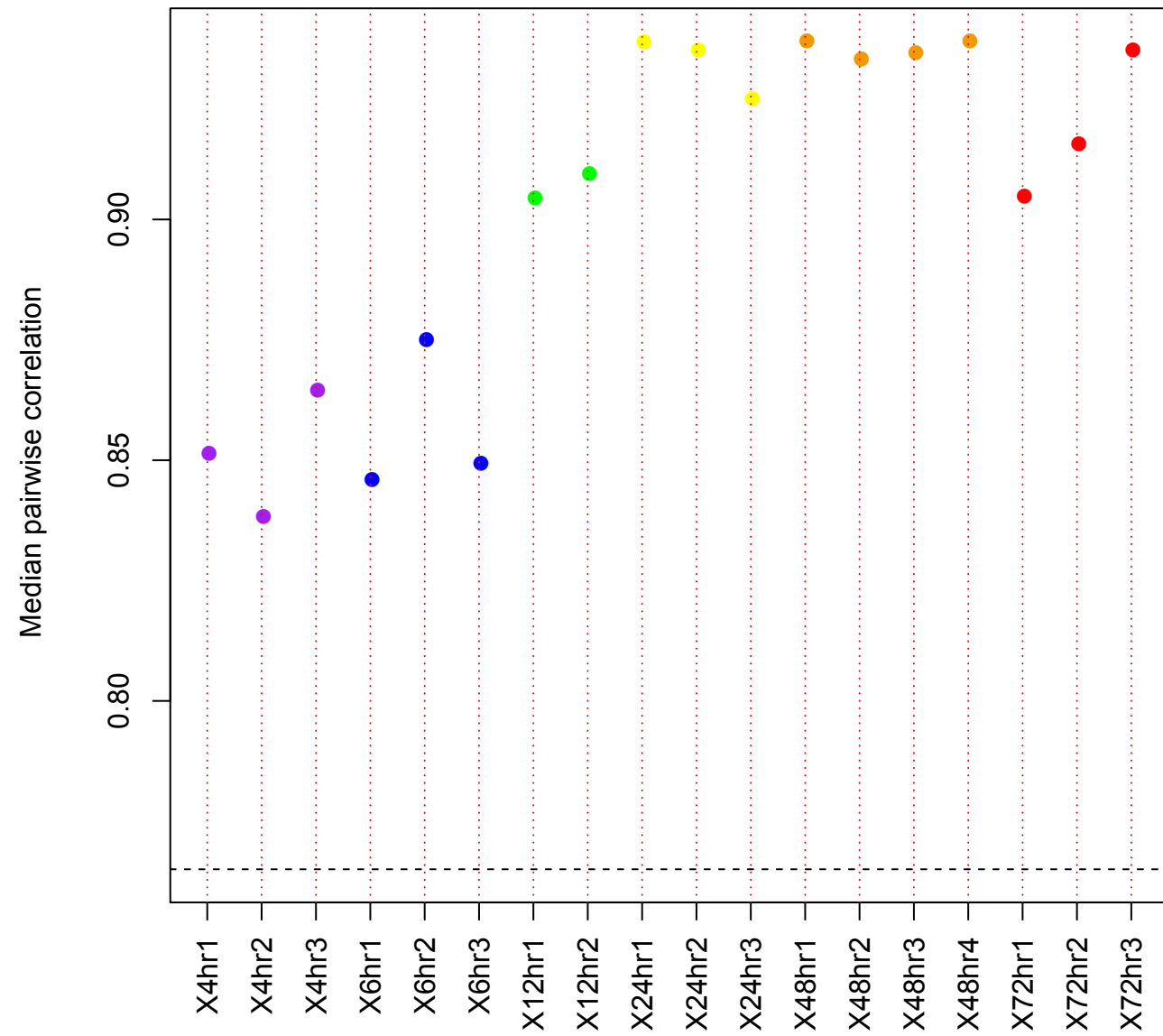

B

Extracellular *T.cruzi* samples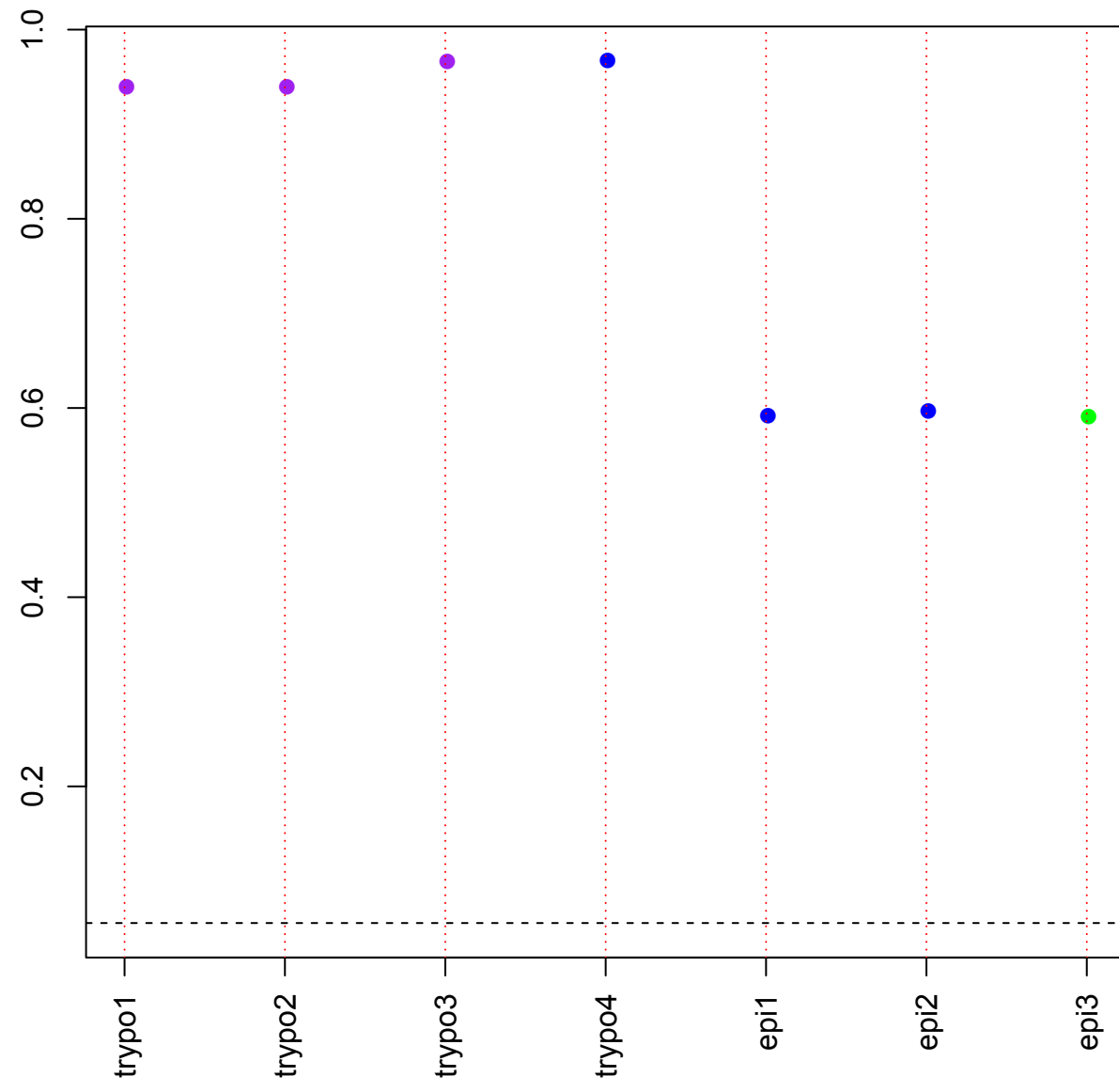

C

Human samples

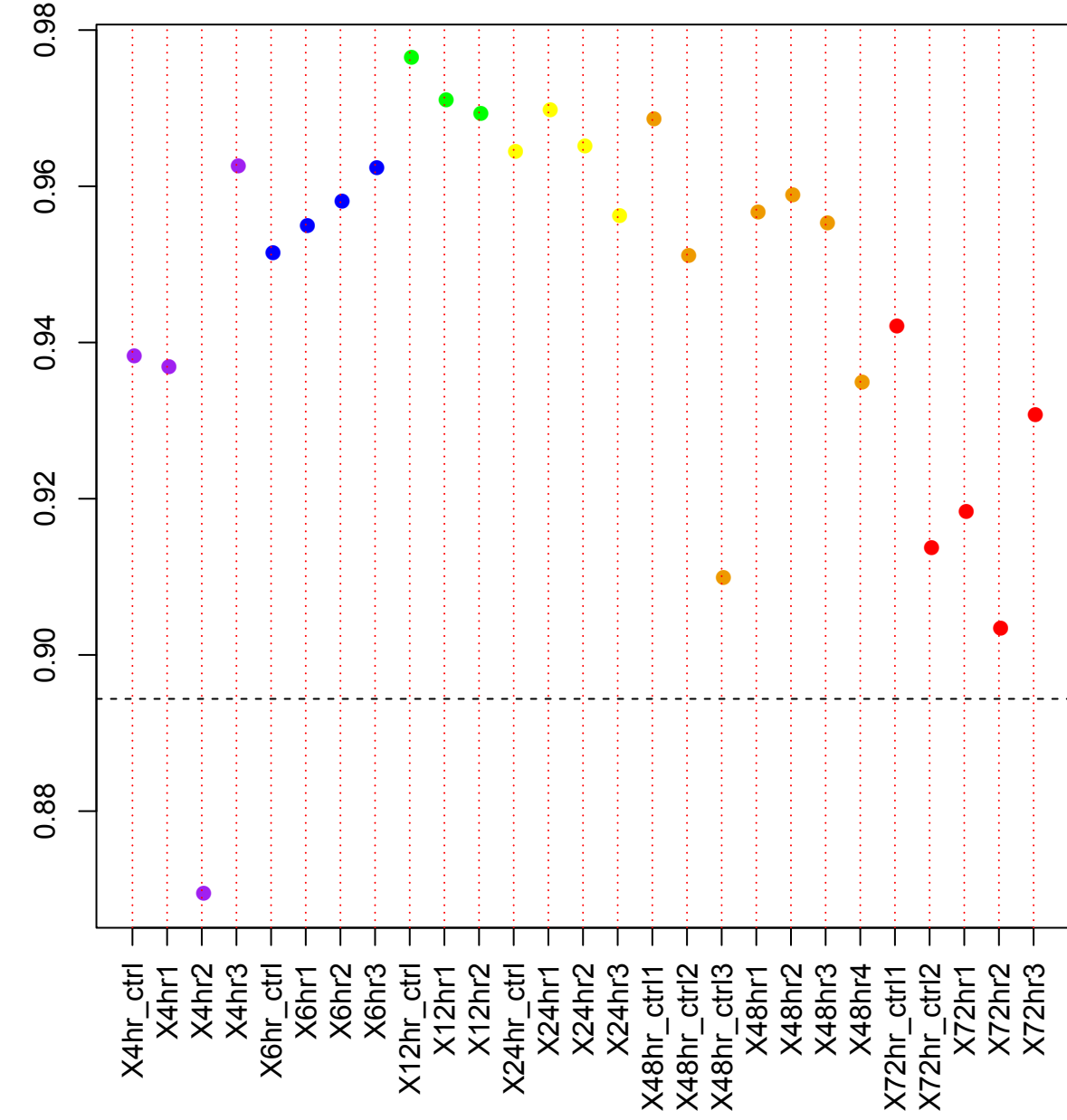

Supplement: S6 Fig — Gene counts were normalized for sequencing library size. The standardized median Pearson correlation between each sample and all other samples was plotted to view the relatedness of samples and identify outliers for (A) intracellular T. cruzi; (B) extracellular T. cruzi and (C) human samples. Letters in the sample name refer to experimental batch. (PDF) [file ppat.1005511.s006.pdf]

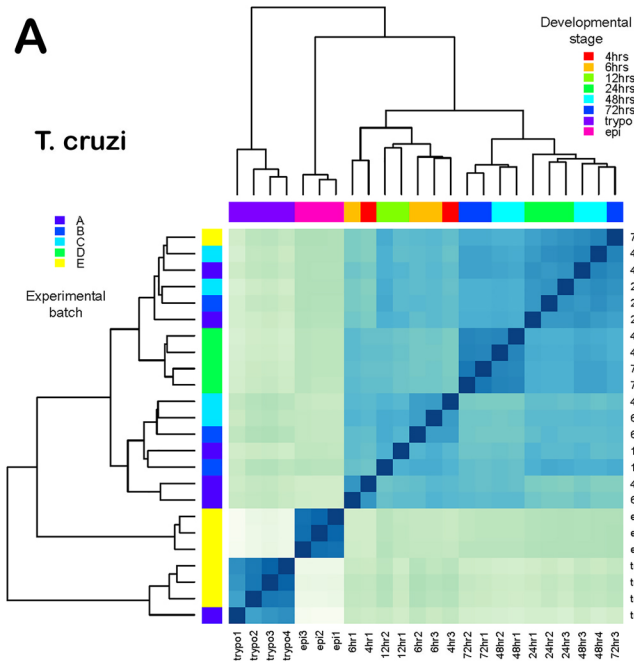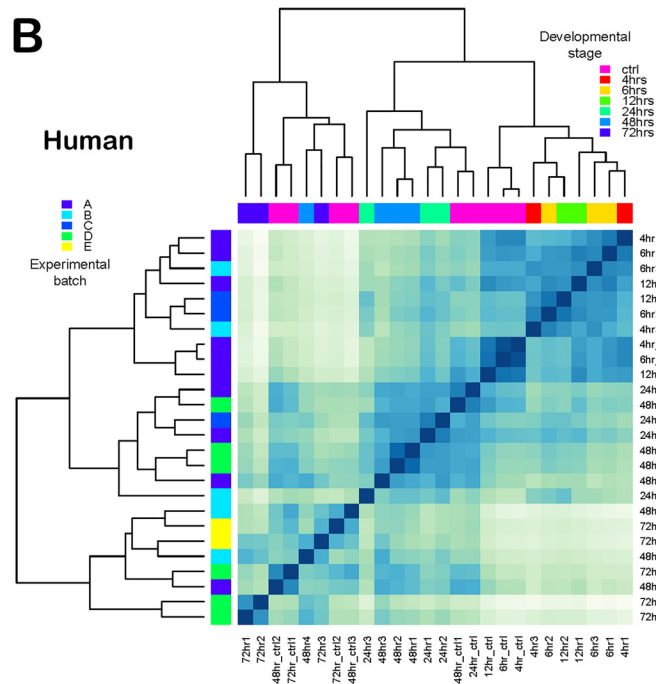

Supplement: S7 Fig — Hierarchical clustering analysis based on Euclidean distance was performed using all (A) T. cruzi or (B) Human genes after filtering for weakly expressed genes, quantile normalization, and inclusion of the batch variable in the statistical model used by Limma. Colors along the top of the heatmap indicate the developmental stage and colors along the left side of the heatmap indicate the batch/experimental date. (PDF) [file ppat.1005511.s007.pdf]

A. *T. cruzi*

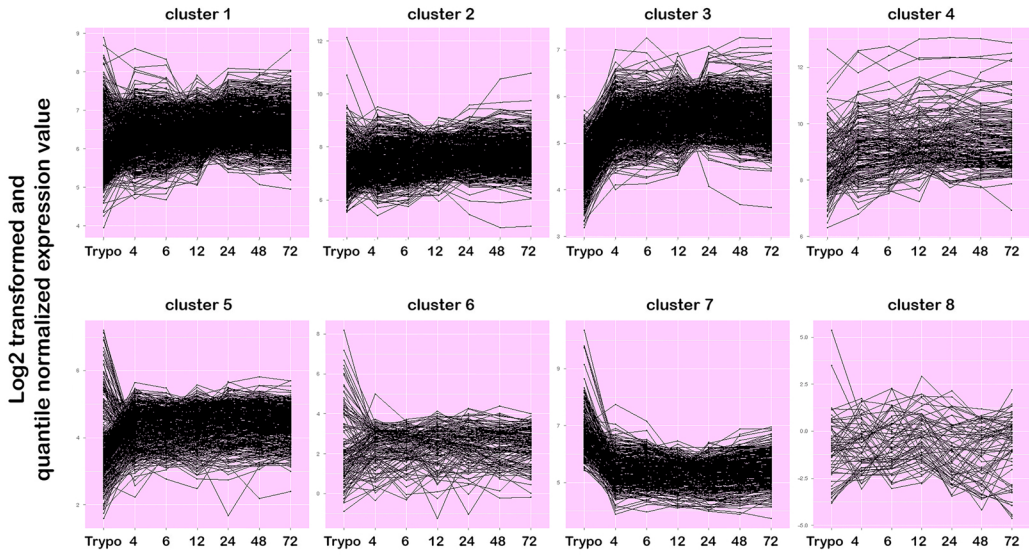

B. Human

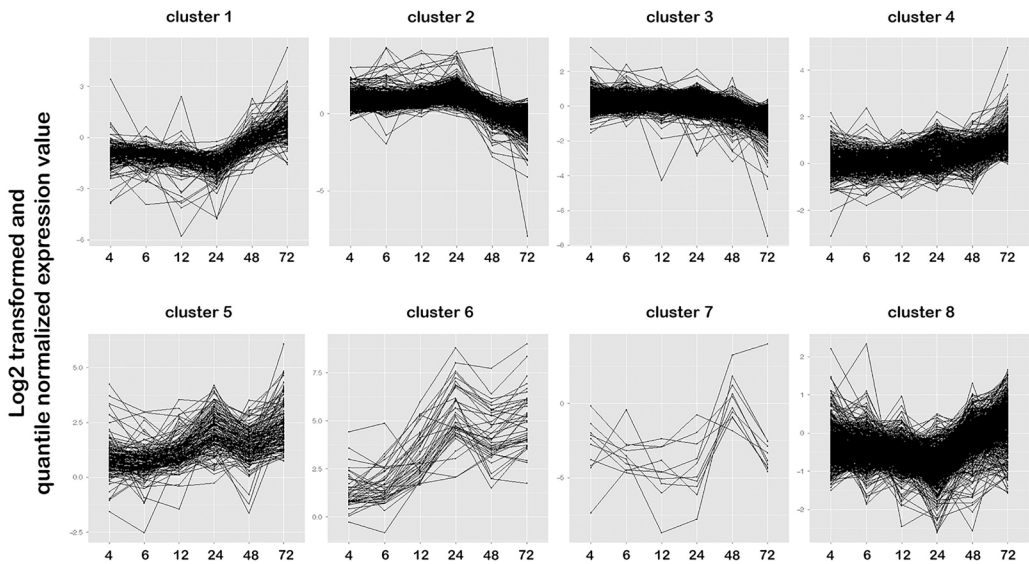

Supplement: S8 Fig — K-means clustering of genes from (A) T. cruzi and (B) human across the intracellular infection course were presented. Log2-tranasformed and quantile-normalized batch-adjusted gene expression values (y-axis) are plotted across the seven conditions (trypo, 4, 6, 12, 24, 48, 72 hpi) for T. cruzi and six time points for human (4, 6, 12, 24, 48, 72 hpi) on the x-axis. Genes included in each of the clusters are listed in S11 Table and S12 Table. (PDF) [file ppat.1005511.s008.pdf]

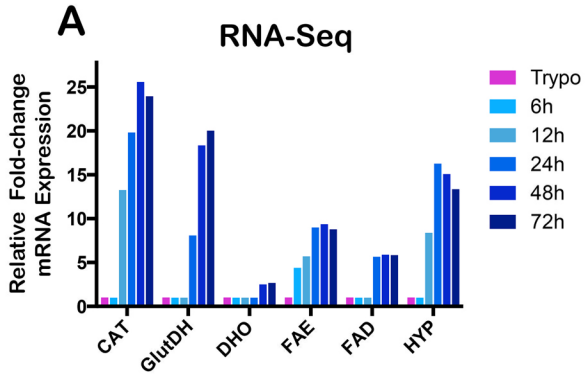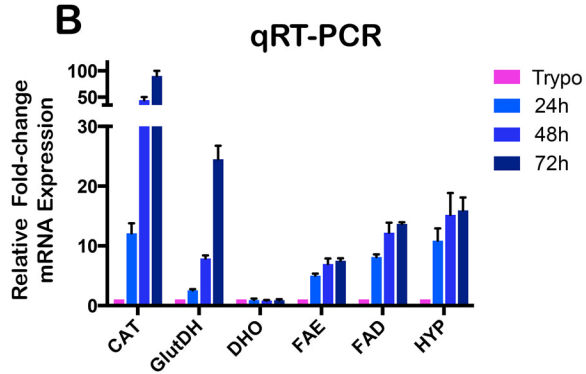

Supplement: S9 Fig — Expression of selected T. cruzi transcripts in intracellular infection stages (6–72 hr post-infection) relative to extracellular trypomastigotes (expression level arbitrarily set to 1). Data derived from RNA-Seq differential expression analysis (A) or qRT-PCR (B) is shown for the following T. cruzi (Y strain) genes: TcCLB.509197.39: Cation transporter (CAT); TcCLB.507875.20: glutamate dehydrogenase (GlutDH); TcCLB.508373.20: dihydroorotase (DHO); TcCLB.506661.30: fatty acid elongase (FAE); TcCLB.511073.10: fatty acid desaturase (FAD) and TcCLB.509767.170: hypothetical protein (HYP). Error bars in (B) represent the mean of duplicate samples. (PDF) [file ppat.1005511.s009.pdf]

**A**Relative mRNA level  
log2 fold-change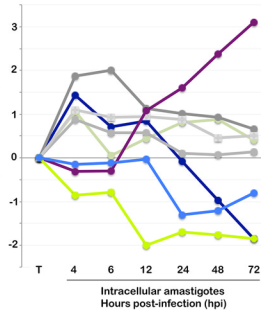**B**Relative mRNA level  
log2 fold-change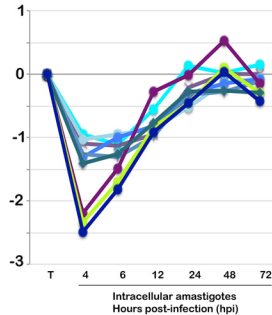

Supplement: S10 Fig — Relative mRNA levels of (A) T. cruzi RNA-binding proteins and (B) flagellar genes that were differentially expressed in at least one of the intracellular amastigote stages (4–72 hpi) as compared to extracellular trypomastigotes (T). (PDF) [file ppat.1005511.s010.pdf]
